# Loci associated with resistance to stripe rust (*Puccinia striiformis* f. sp. *tritici*) in a core collection of spring wheat (*Triticum aestivum*)

**DOI:** 10.1371/journal.pone.0179087

**Published:** 2017-06-07

**Authors:** Kebede T. Muleta, Peter Bulli, Sheri Rynearson, Xianming Chen, Michael Pumphrey

**Affiliations:** 1 Department of Crop and Soil Sciences, Washington State University, Pullman, Washington, United States of America; 2 USDA-ARS, Wheat Health, Genetics, and Quality Research Unit, and Department of Plant Pathology, Washington State University, Pullman, Washington, United States of America; Institute of Genetics and Developmental Biology Chinese Academy of Sciences, CHINA

## Abstract

Stripe rust, caused by *Puccinia striiformis* Westend. f. sp. *tritici* Erikss. (*Pst*) remains one of the most significant diseases of wheat worldwide. We investigated stripe rust resistance by genome-wide association analysis (GWAS) in 959 spring wheat accessions from the United States Department of Agriculture-Agricultural Research Service National Small Grains Collection, representing major global production environments. The panel was characterized for field resistance in multi-environment field trials and seedling resistance under greenhouse conditions. A genome-wide set of 5,619 informative SNP markers were used to examine the population structure, linkage disequilibrium and marker-trait associations in the germplasm panel. Based on model-based analysis of population structure and hierarchical Ward clustering algorithm, the accessions were clustered into two major subgroups. These subgroups were largely separated according to geographic origin and improvement status of the accessions. A significant correlation was observed between the population sub-clusters and response to stripe rust infection. We identified 11 and 7 genomic regions with significant associations with stripe rust resistance at adult plant and seedling stages, respectively, based on a false discovery rate multiple correction method. The regions harboring all, except three, of the QTL identified from the field and greenhouse studies overlap with positions of previously reported QTL. Further work should aim at validating the identified QTL using proper germplasm and populations to enhance their utility in marker assisted breeding.

## Introduction

Stripe rust, caused by *Puccinia striiformis* Westend. f. sp. *tritici* Erikss. (*Pst*), is one of the most devastating diseases of wheat worldwide [1–3]. Historically, stripe rust was mainly a problem of wheat production in areas with cool and wet weather conditions. In recent years, however, the emergence of aggressive and high-temperature-adapted strains of the pathogen has played a significant role in changing the epidemiology of stripe rust to include areas with climates normally considered unfavorable for the disease development [4,5]. These new strains of *Pst* are currently widespread and reduce wheat production on the global scale, heightening a particular concern towards the economic impact of stripe rust on wheat yield and global food security.

Use of genetic resistance is the most effective and economic method for long-term management of stripe rust. Genetic resistance in wheat to stripe rust is categorized as either race-specific or non-race-specific. Race-specific resistance in most cases can be detected at the seedling stage and remains effective at all stages of plant growth, therefore, is often referred to as seedling or all-stage resistance based on “R” genes. Many cultivars with resistance controlled by R genes are usually short-lived due to the emergence of virulent races in the pathogen population. The transient nature of the R-gene mediated resistance has been responsible for the occurrence of many devastating epidemics throughout the world [2,6–9]. On the other hand, non-race-specific resistance genes are mainly expressed at the post-seedling stage of plant development. Therefore, it is often referred to as adult plant resistance (APR). In many cases, APR genes tend to be more durable (remain effective even when used over large acreage for several years) than seedling resistance genes in controlling stripe rust.

In response to “boom-and-bust” cycles, a phenomenon associated with the deployment of single R genes followed by the evolution of matching virulence in the pathogen, much effort has been invested in identifying new sources of durable resistance to stripe rust. Deployment of cultivars carrying combinations of race-specific and non-race-specific resistance genes is a long-term goal for many breeding programs. This approach provides a complex resistance gene landscape against the dynamics of pathogen virulence and should promote durability [7,8,10]. The prospect of deploying resistance gene combinations is being increasingly facilitated by the recent advances in genomics and statistical methods that provide highly effective marker tagging systems and efficient means of genome manipulation [8,11,12].

Employing efficient methods of genetic analysis to facilitate the identification of genomic regions underlying traits of economic and biological importance in a diverse germplasm accessions is a goal for effective utilization of diversity in crop improvement programs. Genome-wide association study (GWAS), a widely used approach for detecting quantitative trait loci (QTL) in plants [13–17], investigates genotype-phenotype correlation by taking advantage of linkage disequilibrium as well as historical recombination present within the gene pool of a species. An additional advantage of GWAS is the detection of QTL with greater resolution from populations of diverse origins, thereby eliminating the need for the time-consuming process of developing mapping populations.

Wheat germplasm collections of the primary gene pool maintained in germplasm banks include landraces, breeding lines and traditional cultivars, which offer access to a diverse range of phenotypes such as disease resistance [18,19]. In some cases, these accessions co-existed with the rust pathogens under a natural evolutionary arms race, which might have resulted in a diversifying selection and accumulation of complex resistance loci [20]. These genetic materials may possess potentially untapped sources of useful genetic resistance owing to their limited use in modern plant breeding programs. The usefulness of wheat landraces and other germplasm maintained in gene banks as a good source of resistance to diseases in wheat have been demonstrated [13,14,16–18,21,22]. Here, we hypothesized that the global collection of the spring wheat accessions maintained by the United States Department of Agriculture-Agricultural Research Service National Small Grains Collection (USDA-ARS NSGC) are useful genetic resources that provide a wide range of diversity for *Pst* resistance. The present study addresses the following three objectives: (1) to assess the diversity of stripe rust resistance in a world-wide collection of spring wheat accessions, (2) to carry out a genome-wide search for single-nucleotide polymorphism (SNP) loci associated with resistance to current *Pst* populations in the Pacific Northwest region of the United States, and (3) to establish relationships between the *Pst* resistance loci identified in this study and previously identified *Yr* genes and QTL.

## Materials and methods

### Wheat germplasm resources

**Fig 1:**
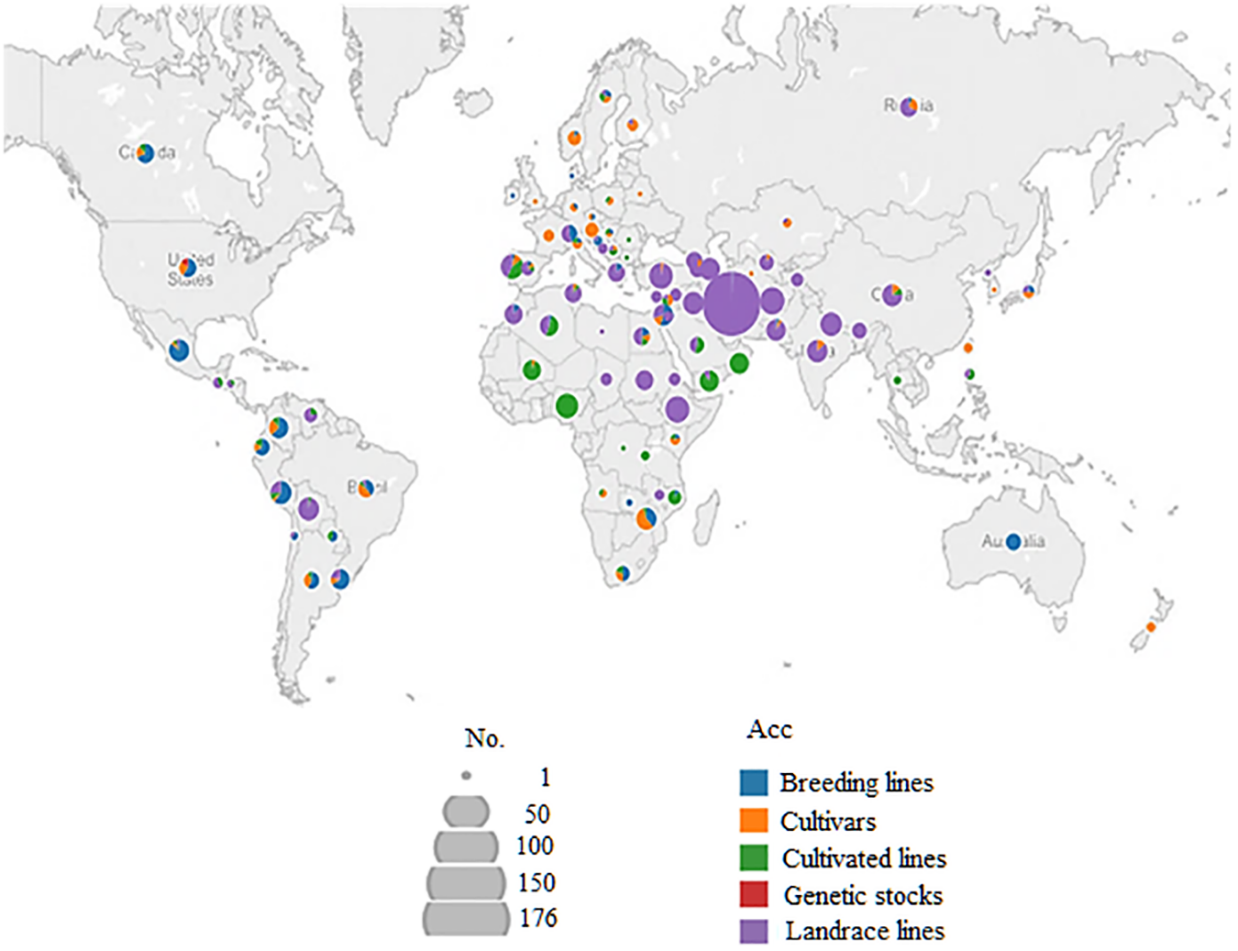
Geographic origin of the 959 spring wheat accessions. The number of accessions (No) from a particular country is indicated by the size of the pie chart. Color within the chart indicates accession type (Acc).

A total of 1,163 spring wheat (*Triticum aestivum* ssp. *aestivum*) accessions were provided by USDA-ARS National Small Grains and Potato Germplasm Research Unit (Aberdeen, Idaho). The accessions were collected from 91 countries in six contents, including Asia (38.8%), Europe (23.2%), Africa (18.9%), South America (14.0%), North America (2.8%), Australia and New Zealand (1.8%) (**Fig 1**). Accessions with unknown origin account for 0.5% of the population. We excluded accessions with greater than 10% missing genotypic data from the final analyses. Genetically redundant accessions were also culled based on Identity-By-Descent (IBD) kinship analysis, and represented by only one individual accession. Accordingly, a total of 959 non-duplicate accessions were used for the analyses.

### Phenotypic trait evaluation

#### Field-based resistance screening

Adult plants of the accessions were tested for response to *Pst* infection under field conditions at two locations in the Pacific Northwest (PNW) of the U.S. during 2012–2014 crop seasons. The locations were Mount Vernon, WA (48° 25’ 12”N; 122° 19’ 34”W), a high rainfall area located west of the Cascade Mountain range; and Pullman, WA (46° 43’ 59'' N; 117° 10’ 00”W), a semi-arid wheat belt area located east of the Cascade Mountain range. The nursery locations are subject to high disease pressure on an annual basis, but vary in *Pst* populations and weather patterns. The highly susceptible cv. 'Avocet S' was planted every 20 rows and on each side of the plot to ensure uniform disease pressure across the experimental plots. Infection type (IT) to stripe rust was recorded using a 0–9 scale [23]. The SEV and IT data collected from Pullman in 2013 were excluded from subsequent analyses due to low and uneven disease development during that particular season. Phenotypic data are available at the Triticeae Toolbox https://triticeaetoolbox.org/wheat/.

#### Seedling resistance screening

Races PSTv-14, PSTv-37 and PSTv-40, representing the prevalent races of *Pst* in the PNW and across the U.S. [24], were used to screen seedlings of the accessions under controlled conditions in a greenhouse. The virulence/avirulence formulae of the three races are given in **S1 Table**. The seedlings were evaluated following the standard procedure described in [24].

### DNA extraction and genotyping

Genomic DNA was extracted from leaf tissues of 10–15 days old seedlings of the 1,163 spring wheat core collection at the USDA-ARS Small Grains and Potato Germplasm Research Unit. DNA extractions were performed using the CTAB protocol [25]. The DNA was precipitated by adding isopropanol, followed by washing of the pellet with ice-cold 70% ethanol, and resuspension in 200 μL of Tris HCl ethylenediaminetetraacetic acid (pH 8.0).

Whole genome profiling of the accessions was performed using the Illumina iSelect 9K wheat assay at the USDA-ARS Wheat Genotyping Laboratory, Fargo, ND. Genotype calling of the original Illumina data was carried out using Illumina’s GenomeStudio v2011.1 software to optimize the SNP call rates for misclassification and ambiguous clusters. After removing SNPs with low-quality clustering and those with minor allele frequency (MAF) less than 5%, a total of 5,619 high quality SNP markers with genetic map information were used for GWAS analyses. The genetic positions of the SNP markers were based on the wheat 9K SNP consensus map [26]. Genotypic data are available at the Triticeae Toolbox https://triticeaetoolbox.org/wheat/.

For controls and validation, the collection was screened with molecular markers for the known stripe rust resistance genes. These include, *Lr34*/*Yr18*/*Pm38* (KASP wMAS000003) (http://maswheat.ucdavis.edu/protocols/Lr34/index.htm), *Lr37*/*Yr17*/*Sr38* (marker Ventriup-LN2) (http://maswheat.ucdavis.edu/protocols/Lr37/index.htm), *Lr67*/*Yr46* (Kasp856) [27], *Yr30*/*Sr2* (KASP marker wMAS000005) (http://maswheat.ucdavis.edu/protocols/Sr2/index.htm) and *Lr68* (CAPS marker cs7BLNLRR) [28] (http://maswheat.ucdavis.edu/protocols/Lr68/index.htm). The MAF for each control marker, except for *Lr37*/*Yr17*/*Sr38* and *Yr30*/*Sr2*, were greater than 5%, and were thus included in the GWAS and linkage disequilibrium analyses.

### Population structure and linkage disequilibrium

Population structure was investigated using the Bayesian model-based clustering algorithm implemented in the STRUCTURE software version 2.2.3 [29]. A set of 425 SNP markers distributed across wheat genetic map with an inter-marker distance of >10 cM were used to infer population structure in the germplasm panel. A burn-in of 50,000 iterations and 100,000 Monte Carlo Markov Chain (MCMC) replicates were set to determine K values (number of subpopulations) in the range of 1 to 10. For each K, five independent runs were carried out. The Evanno method [30] was used to determine the likely number of subpopulations using STRUCTURE HARVESTER [31]. Principal component analysis (PCA) was also performed using JMP GENOMICS software (JMP^®^, Version 6.1. SAS Institute Inc., Cary, NC) to further explore the pattern of population sub-structuring and compare with the STRUCTURE results.

Pairwise measures of linkage disequilibrium (LD) between pairs of SNP markers were estimated using JMP GENOMICS version 6.1. LD was estimated as squared allele frequency correlations (*r*^*2*^) between pairs of intra-chromosomal SNPs with known chromosomal position and minor allele frequency (MAF) > 0.05. To determine the average pattern of genome-wise LD decay over genetic distance, a scatterplot of *r*^*2*^ values against the corresponding genetic distance between markers was constructed. The second-degree locally weighted polynomial regression (LOESS)-based curve was fitted to estimate the extent of LD decay [32].

### Estimation of BLUPs and variance components

Best linear unbiased predictors (BLUPs) were estimated for each accession at each location using the phenotypic trait values available for each test year as a predictor. BLUPs were estimated using a mixed effects model by fitting years as a fixed effect and accessions as a random effect using the lme4 package [33] in R (R Development Core Team). The mixed model was also used to estimate variance components for genotypes, environments, and genotype by environment interactions. Trait heritability (*h*^*2*^) estimates were calculated using the Restricted Maximum Likelihood (REML) method [34].

### Association analysis

Marker-trait association was performed using the compressed mixed linear model (cMLM) method [35,36] implemented in GAPIT (Genomic Association and Prediction Integrated Tool) in R environment [37]. In the mixed model analysis, the 959 × 959 kinship matrix and the first two principal components determined by PCA were used as covariates to control spurious associations due to cryptic relatedness and population structure, respectively. A marker-wise association probability value of *P* <0.01 in at least two of the five test environments was used to declare significant marker-trait associations (MTAs). To minimize the chance of false positive MTAs, associations significant at genome-wide adjusted *P* <0.1 based on the False Discovery Rate (FDR) multiple correction method [38] were also identified. Inter-marker distance ≥ 2.5 cM, which is genomic distance below which genome-wide LD was predicted to decay, was used to establish QTL confidence interval (CI) of ±2.5 cM. Among the adjacent SNPs, the QTL-tagging SNP was selected as the one that showed the strongest *P* value.

Stepwise regression analysis was also performed to identify the best combinations of significant alleles explaining the variation for stripe rust resistance in the germplasm panel. Stepwise regression also facilitates the selection of markers that have a major effect in a QTL region and simultaneously exclude other markers in LD with the major marker within the confidence interval of the QTL region. A significant *P* value threshold of 0.1 and 0.05 was used in the stepwise regression analysis for the inclusion and exclusion of the markers in the analysis, respectively. Finally, multiple linear regression analysis was performed to assess the amount of phenotypic variation explained by the significant markers. The markers were used as the independent variable and BLUP values of IT and SEV from the field trials were included as the response variable.

### Relationship between number of favorable alleles and response to stripe rust

Pearson correlation analyses were performed to assess the relationship between the number of favorable alleles of the significant QTL-tagging SNPs accumulated in a single accession and stripe rust resistance. Favorable and alternate alleles for the QTL-tagging markers that were significant at *P* < 0.01 in at least two environments were coded as 1 and 0, respectively, and the cumulative numbers of favorable and the alternate alleles were counted for each accession for use in the correlation analysis.

### Ethics statement

No specific permits were required for the described field studies. No specific permissions were required for these locations/activities. The study locations are not privately-owned or protected in any way. In addition, the field studies did not involve endangered or protected species.

## Results

### Estimates of variance components and trait heritability

**Fig 2:**
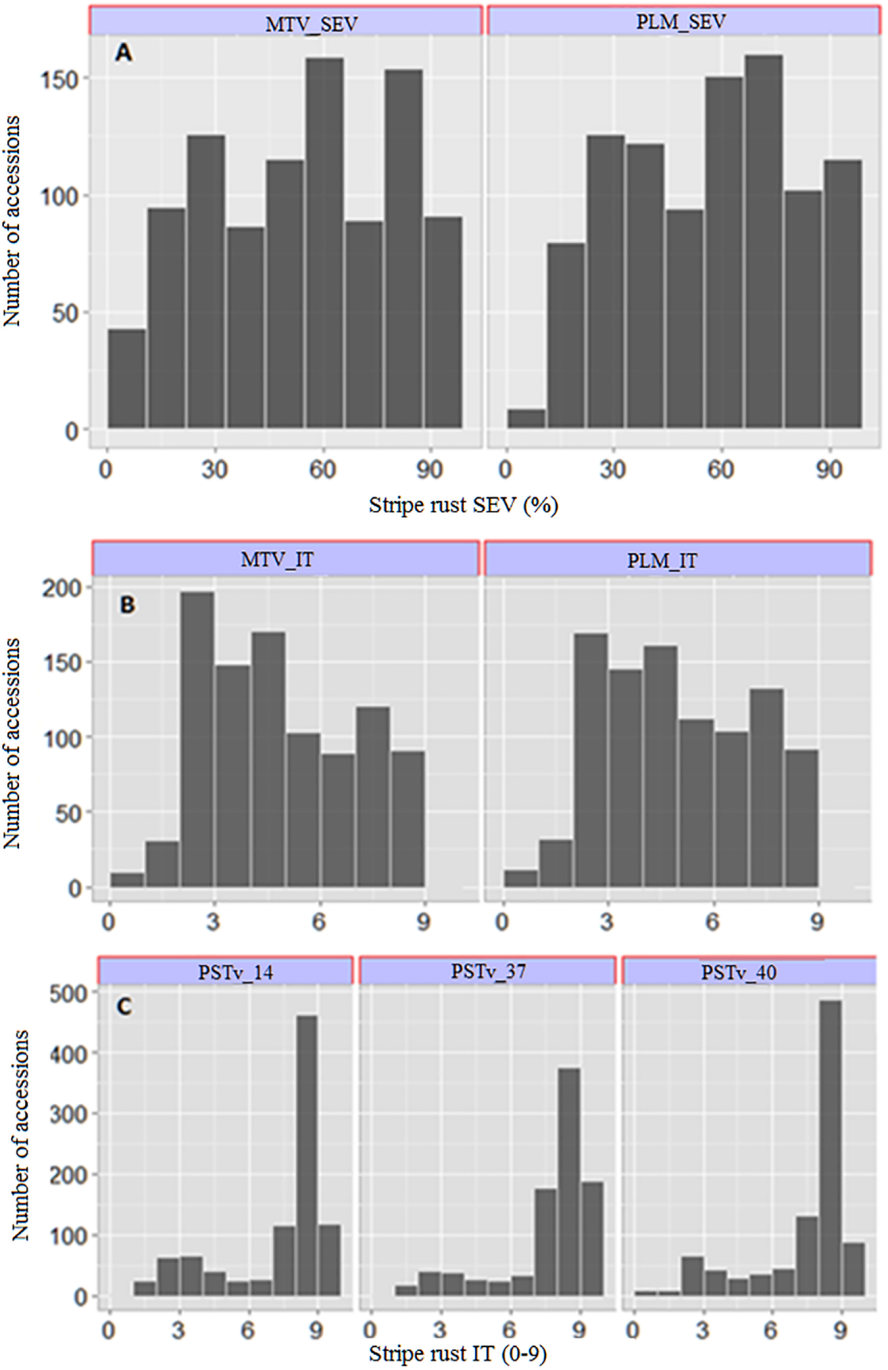
Frequency distribution of the response to the accessions to stripe rust. A) Best linear unbiased prediction (BLUP) values of the infection type (IT) responses of adult plants under field condition, B) BLUP values of the severity (SEV) responses of adult plants under field condition, and C) seedling resistance screening against three *Puccinia striiformis* f. sp. *tritici* races (PSTv_14, PSTv_37 and PSTv_40) under greenhouse experiments. MTV_SEV = Mount Vernon severity, MTV_IT = Mount Vernon infection type, PLM_SEV = Pullman severity, PLM_IT = Pullman infection type.

Mean responses of the accessions to stripe rust as well as estimates of variance components and broad sense heritability are summarized in **Table 1**. The mixed linear model analysis using the SAS procedure PROC MIXED revealed highly significant differences among the genotypes and genotype × environment interactions for SEV and IT (*P* <0.0001). Using the BLUP values to group the accessions into categories based on IT, 18% of the accessions displayed high resistance reactions (IT = 0–3), while 24% of the accessions were considered highly susceptible (IT = 7–9) across all environments. The remaining 58% of the accessions showed either an intermediate reaction (IT = 4–5) across all environments, or variable responses at different test environments (**Fig 2**). Heritability (*H*^*2*^) values for IT were 0.84 and 0.86 in Pullman and Mount Vernon, respectively, while the heritability estimates for SEV were 0.80 in Pullman and 0.90 in Mount Vernon. Consistent with the high heritability estimates, we also observed highly significant correlations for IT and SEV among the five test environments (**S2 Table**).

**Table 1:** Means and variance components of infection type (IT) and severity (SEV) of stripe rust in the 959 global collection of spring wheat accessions.

Trait	Mount Vernon		Pullman		Across all environments	
	IT	SEV	IT	SEV	IT	SEV
Mean	5.0	53.5	4.9	55.4	4.9	54.3
Max.	9.0	100.0	9.0	100.0	9.0	100.0
Min.	0.0	0.0	0.0	0.0	0.0	0.0
σ^2^_G_	5.0^***^	750.2^***^	5.09^***^	697.5^***^	5.1^***^	744.2^***^
σ^2^_E_	0.4^ns^	7.3^ns^	0.01^ns^	38.6^ns^	0.2^ns^	14.3^ns^
σ^2^_GXE_	0.9^***^	240.4^***^	0.95^***^	298.4^***^	-	248.5^***^
σ^2^_error_	1.1^ns^	1.0^ns^	1.02^ns^	1.0^ns^	1.9^***^	1.0^ns^
Heritability	0.86	0.90	0.84	0.80	0.92	0.93

IT = stripe rust infection type; SEV = stripe rust severity; σ^2^_G_ = genotype variance, σ^2^_E_ = environment variance, σ^2^_G×E_ = genotype × environment variance, σ^2^_erro**r**_ = error variance, ns = not significant

^***^ *P* < 0.0001.

### Population structure and linkage disequilibrium

**Fig 3:**
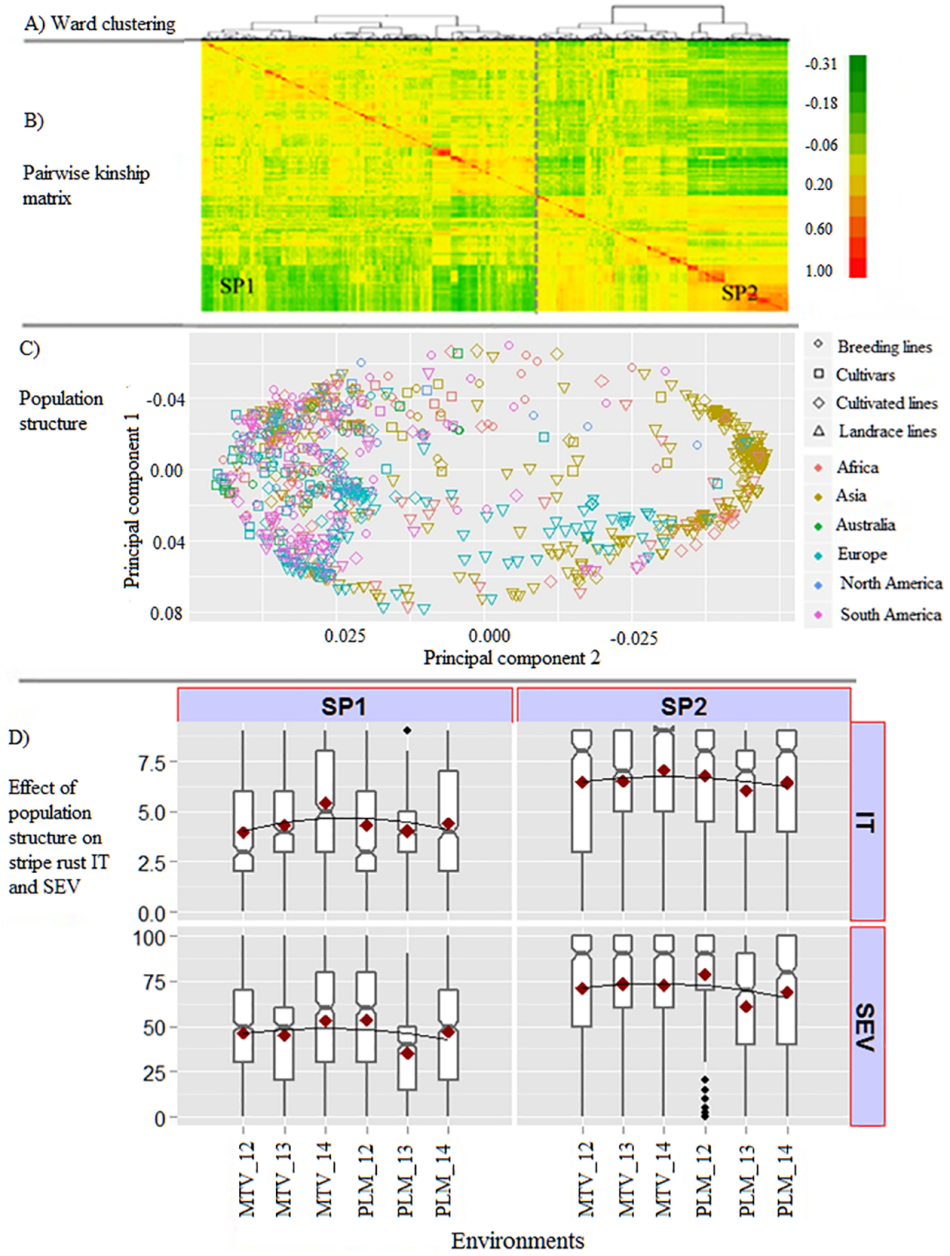
Population structure and its relationship to stripe rust resistance. A) Dendrogram based on Ward clustering of the spring wheat core collection, B) pairwise kinship matrix depicting clustering of the accessions based on identity-by-decent (IBD), C) population structure based on principal component analysis (PCA). Both genetic relatedness and principal component analyses grouped the accessions into two subpopulations, Subpopulation 1 (SP1) and Subpopulation 2 (SP2). Clustering pattern based on PCA also explained geographic origin and improvement status of the spring wheat core collection. D) Effect of population structure on stripe rust infection type (IT) and severity (SEV). Box plots show trait distribution and compare the levels of stripe rust between the two subpopulations. MTV_12 = Mount Vernon 2012, MTV_13 = Mount Vernon 2013, MTV_14 = Mount Vernon 2014, PLM_12 = Pullman 2012, PLM_13 = Pullman 2013, PLM_14 = Pullman 2014.

Both STRUCTURE and PC analyses grouped the accessions into two major clusters (**Fig 3**, **S1 Fig**). Furthermore, the distance-based Fast Ward hierarchical clustering also revealed a similar grouping pattern. Population structure was also evident when the accessions were grouped by geographic origin and improvement status (ACIMPT). The first sub-population (sub-population 1) composed of accessions mainly from Europe, North America and South America, while the second sub-population (sub-population 2) constituted landraces collected from Asia. **Fig 3 (C)** depicts a two dimensional scatterplot of the first two PCs of the spring wheat accessions superimposed with geographic origin and improvement status of the accessions. A significant correlation (*P* <0.001 and *r* ranging from 0.11 to 0.30) was observed between the population sub-clusters and response to stripe rust resistance, justifying the use of a GWAS model accounting for population structure in the panel.

**Fig 4:**
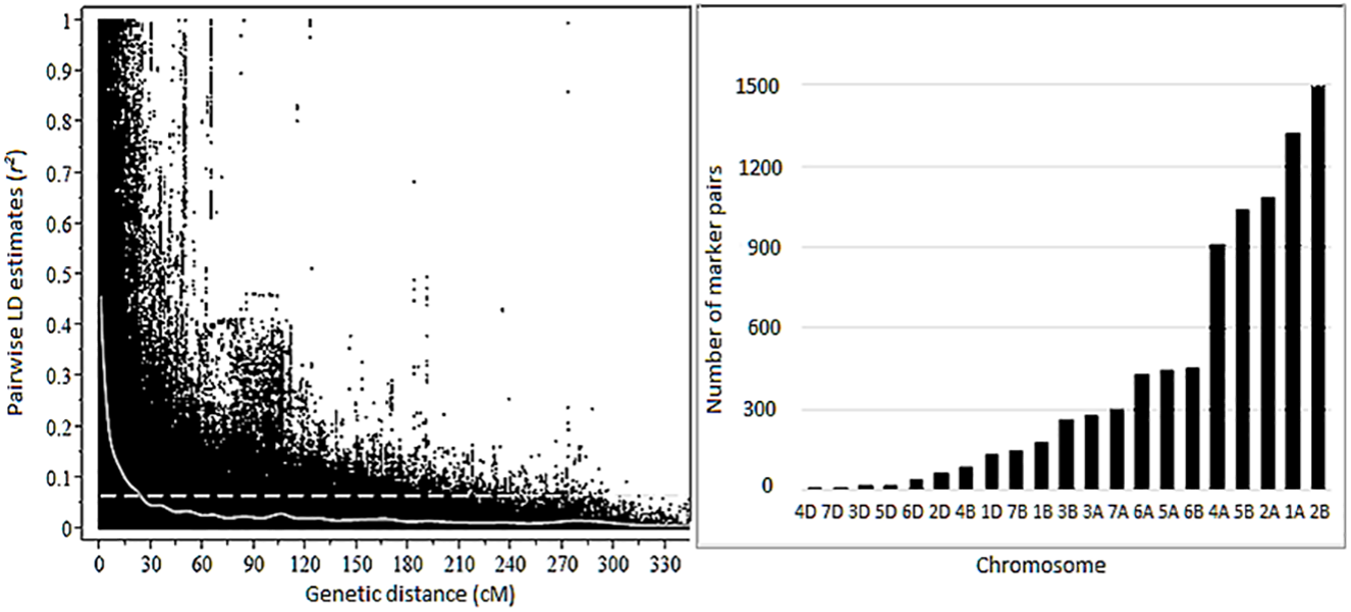
Patterns of genome wide linkage disequilibrium (LD) in the germplasm panel. A) Scatter plot of average LD (*r*^*2*^) as a function of genetic distance between markers, B) chromosome-wise distribution of the number of marker pairs that showed LD due to physical linkage (B).

Genome-wide LD was predicted to decay below the critical *r*^*2*^ = 0.36 at an inter-marker distance of 2.5 cM (**Fig 4**). Average *r*^*2*^ value was 0.55 for the completely linked marker pairs (markers with zero inter-marker distance). Among the non-completely linked marker pairs whose genetic distance was less than 1 cM, the *r*^*2*^ value decreased to 0.3. This indicates a decrease in *r*^*2*^ value to half of its initial value within 1 cM. Among the marker pairs in LD due to linkage (*r*^*2*^ > 0.36), chromosome 2B contained the highest percentage (17.2%) of these markers, while chromosome 4D contained the lowest percentage (less than 1%). The proportion of marker pairs in the A, B and D genomes that are in LD at *r*^*2*^ >0.36 were 55.3, 41.7 and 7.0%, respectively (**Fig 4**).

### Marker-trait association and annotation

#### Field-based resistance to stripe rust

GWAS identified a total of 130 SNPs associated with resistance to stripe rust at adult plant stage when filtering loci significant at marker-wise *P* <0.01 in at least two of the five test environments. Among the 130 significant SNPs, multiple co-locating SNPs were assigned to a unique QTL region based on the following approaches. First, a confidence interval for assigning the putative resistance loci were determined based on a threshold of the inter-marker distance (cM) at which LD has decayed below the critical *r*^*2*^ value. Among the multiple SNPs within a confidence interval of a QTL, the SNP that showed the strongest association and significant in the stepwise regression analysis was selected as the QTL-tagging SNP. Of the 130 significant SNPs identified, 70 fit into the stepwise regression model and also corresponded with the SNPs that showed the strongest association (smallest *P* value) in the GWAS test; each represented a unique QTL region, which was determined based on the confidence interval for determining putative resistance loci (±2.5 cM around the QTL-tagging SNP) (**S3 Table**). The 70 QTL-tagging SNPs explained 51% and 52% of the total variation for stripe rust IT and SEV, respectively. QTL regions were detected on all chromosomes except on 3D and 7D. Detailed information from the GWAS and stepwise regression tests for the 70 QTL is presented in **S3 Table** and **S4 Table**, respectively.

**Fig 5:**
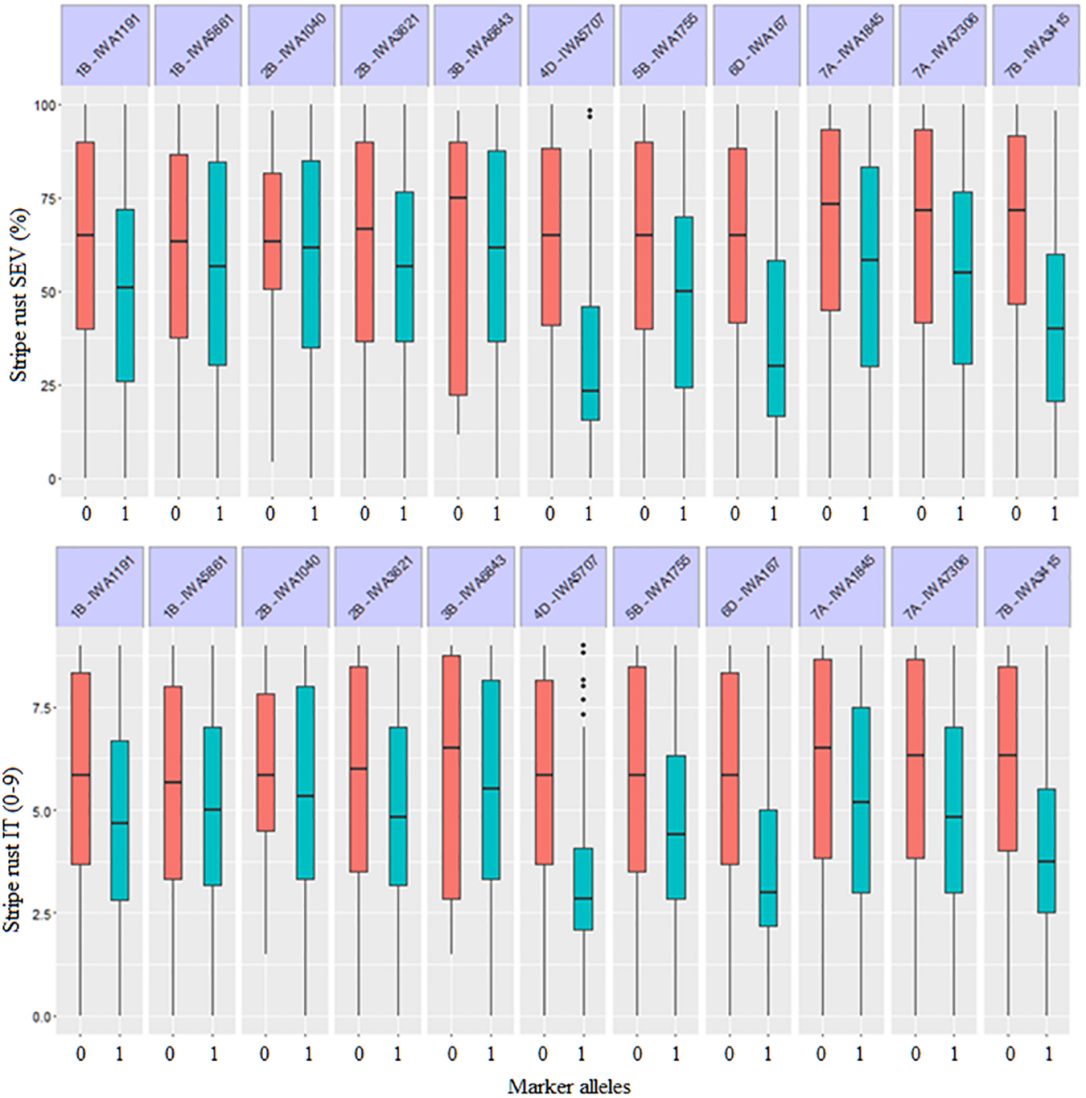
Allelic effects of the 11 highly significant markers on BLUP values of stripe rust severity (upper) and infection type (lower). Blue colored boxes indicate effect due to the resistance associated alleles, while the red colored boxes indicated effect due to the unfavorable allele.

Eleven of the 70 QTL-tagging SNPs were significant at FDR-adjusted *P* <0.10. The 11 genomic regions were represented by SNPs *IWA1191* and *IWA5861* (on chromosome 1B at 23.7 and 94.2 cM respectively), *IWA3621* and *IWA1040* (on chromosome 2B at 114.3 and 210.2 cM, respectively), *IWA6843* (on chromosome 3B at 111.6 cM), *IWA5707* (on chromosome 4D at 25.7 cM), *IWA1755* (on chromosome 5B at 130.4 cM), *IWA167* (on chromosome 6D at 16.8 cM), *IWA7306* and *IWA1845* (on chromosome 7A at 3.9 and 42.5 cM, respectively) and *IWA3415* (on chromosome 7B at 164.9 cM). The resistance alleles of the 11 genomic regions showed average allelic effect of reducing stripe rust responses ranging from 0.3 to 1.8 for IT and 2.2 to 23.0 for SEV (**Fig 5**). Collectively, these QTL explained 24% of the observed variation for both IT and SEV. Detailed information on the 11 QTL is summarized in **Table 2.** The integrated map developed by Maccaferri et al. [17] was used to establish the relationship between the 11 QTL with significant genome-wide associations (FDR-adjusted *P* <0.1) with previously mapped *Yr* genes and QTL. Accordingly, any *Yr* genes and QTL previously mapped within the ±2.5 cM confidence interval of the 70 QTL detected in the present study were identified and are presented in **Table 2.** QTL with confidence intervals not overlapping with that of previously reported *Yr* gene or QTL were considered to represent newly-discovered *Pst* resistance loci. Two of the 11 loci identified from the field studies (*IWA1755* on 5BL and *IWA7306* on 7AS) were mapped far from previously identified *Pst* resistance genes and QTL and likely represent new resistance loci.

**Table 2:** Genomic regions significantly associated with field-based resistance to stripe rust infection type (IT) and severity (SEV) in the 959 spring wheat accessions based on marker-wise *P* value <0.01 in at least two of the five environments and false-discovery-rate (FDR) adjusted *P* values < 0.1 in at least one environment.

Chr.	QTL-tagging SNP^1^	Alleles^2^	Associated SNP^3^	Position^4^ (cM)	MAF	P values (-log)^5^		Environments with marker-wise significant MTA (IT/SEV)	Previously mapped *Yr* Gene/QTL^6^	References
						IT	SEV			
1B	*IWA1191*	T/C	*IWA3155*, *IWA7480*	23.65	0.18	2.3	2.6	PLM12, MTV12, MTV13, MTV14/PLM12, MTV12, MTV14	*Yr15*, *Yr24*, *YrAlp*, *Yr64*, *YrCH52*, *Yr65*, *QYr*.*caas-1BL*.*1RS_SHA3/CBRD*	Cheng et al., 2014, Ma et al., 2001, Ren et al., 2012
1B	*IWA5861*	T/C	*IWA3017*	94.2	0.23	2.9	2.8	PLM14, MTV14/ MTV13, PLM14	*QYr*.*sun-1B_CPI133872*, *QYr*.*sun-1B_Kukri*, *QYr*.*sun-1B_Wollaroi*, *QYr*.*cim-1BL_Francolin*, *QYr*.*tam-1B_Quaiu*, *QYr-1B_Saar*	Zwart et al., 2010, Bariana et al., 2010, Bansal et al., 2014, Lan et al., 2014, Basnet et al., 2014, Lillemo et al., 2008
2B	*IWA3621*	A/G	*IWA2977*, *IWA4983*, *IWA4984*, *IWA5149*, *IWA6875*, *IWA6818*	114.3	0.31	2.6	2.5	PLM12, MTV12, MTV14, MTV13/ PLM12, MTV12, PLM14, MTV14	*YrKK*, *QYr*.*inra-2B*.*1_Camp Remy*, *QYr*.*ucw-2B_UC1110*, *QYr*.*tam-2BL2_TAM111*, *QYr-2B_Opata 85*, *Qyrlo*.*wpg-2B_Louise*, *QYrid*.*ui-2B*.*2_IDO444*	Li et al., 2013, Mallard et al., 2005, Lowe et al., 2011, Basnet et al., 2014, Boukhatem et al., 2002, Carter et al., 2009, Chen et al., 2012
2B	*IWA1040*	A/G	*IWA8266*	210.2	0.11	2.9	2.7	PLM12, MTV12, PLM14, MTV14/ PLM12, MTV12, MTV13, PLM14, MTV14	*Yr43*, *Yr44*, *Yr53*, *QYraq*.*cau-2BL_Aquileja*	Xu et al., 2013, Guo et al., 2008
3B	*IWA6843*	A/G	*-*	111.6	0.44	2.8	3.1	PLM12, MTV12, MTV13, MTV14/ PLM12, MTV12, MTV13, PLM14	*QYr*.*cim-3B_Pastor*, *QYr*.*inra-3Bcentr_Renan*	Rosewarne et al., 2012, Dedryver et al., 2009
4D	*IWA5707*	T/C	*IWA6277*	25.7	0.07	2.8	2.8	PLM14, MTV14/ PLM14, MTV14	*Yr46/Lr67*	Herrera-Foessel et al., 2011
5B	*IWA1755*	A/G	*IWA6627*	130.4	0.18	2.6	2.8	PLM12, MTV12, PLM14, MTV14/ MTV13, PLM14	-	-
6D	*IWA167*	A/G	*IWA2808*, *IWA3624*	16.8	0.11	2.7	2.9	PLM12, MTV12, MTV13, PLM14/ PLM12, MTV12, MTV13, PLM14	-	-
7A	*IWA7306*	A/G	*-*	3.9	0.47	2.8	2.5	MTV12, MTV13, PLM14/MTV13, PLM14	QYr.sun-7A_CPI133872, QYr.caas-7A_Jingshuan16	Rosewarne et al., 2012, Crossa et al., 2007
7A	*IWA1845*	T/C	*IWA2513*	42.5	0.34	2.91	2.54	PLM12, MTV12, MTV13, MTV14/PLM12, MTV12, MTV13, MTV14	*QYr*.*sun-7A_CPI133872*, *QYr*.*caas-7A_Jingshuan16*	Zwart et al., 2010, Ren et al., 2012
7B	*IWA3415*	A/G	*IWA3416*	164.9	0.27	3.13	3.85	PLM12, MTV12, PLM14, MTV14/ PLM12, MTV12, MTV13, PLM14	*Yr59*, *YrC591*, *Yr52*, *Yr67*, *YrZH84*	Zhou et al., 2014b, Xu et al., 2014, Li et al., 2006

^1^SNP index from the wheat 9K iSelect assay.

^2^Underline indicates favorable allele.

^3^Other significant SNPs identified within the confidence interval of the QTL.

^4^Based on the consensus map of the wheat 9K iSelect assay by Cavanagh et al. [26].

^5^*P* values based on BLUP values of stripe rust IT and SEV across all environments.

^6^QTL/gene previously mapped within the confidence interval of the putative QTL identified in this study

MAF—Minor allele frequency.

PLM12 = Pullman 2012, PLM14 = Pullman 2014, MTV12 = Mount Vernon 2012, MTV13 = Mount Vernon 2013, MTV14 = Mount Vernon 2014.

#### Seedling stage resistance

Seven loci were significantly associated with IT response to the three races of *Pst* at seedling stage (**Table 3**). These SNPs were detected on chromosome 4B (*IWA2194* mapped at 49.4 cM), 5A (*IWA2145* and *IWA1258* mapped at 19.7 cM and 235.1 cM, respectively), 5B (*IWA7815* mapped at 167.8 cM), 6A (*IWA2129* mapped at 212.2 cM) and 7B (*IWA312* and *IWA2770* mapped at 76.1 cM and 246.5 cM, respectively). SNPs *IWA2194*, *IWA1258*, *IWA2129* and *IWA2770* were also identified in GWAS of field resistance to *Pst* at the nominal probability of association. The putative resistance loci represented by *IWA2194*, *IWA2145*, *IWA7815* and *IWA312* were effective to the three races of *Pst*. Six of the seven genomic regions significant for conferring seedling resistance to stripe rust were mapped closely to known resistance genes and QTL, while one (*IWA2145* on chromosome 5A) was mapped far from previously identified *Pst* resistance genes and QTL.

**Table 3:** Genomic regions significantly associated with seedling resistance to stripe rust in the 959 global collection of spring wheat accessions based on FDR adjusted *P* values <0.1.

Chr.	QTL-tagging SNP^1^	Alleles^2^	Associated SNP^3^	Position^4^ (cM)	MAF	*P* values (-log)^5^			Significant association under field condition		Previously mapped *Yr* Gene/QTL	References
						PSTv-14	PSTv-37	PSTv-40	*P* values	Environments		
4B	*IWA2194*	A/C		49.4	0.38	3.6	3.1	**4.6**	****	MTV14, PLM12, MTV12, MTV14, PLM14	*QYr-4B_Sachem*, *QYr*.*ufs-4B_Palmiet* *Cappelle*, *QYr*.*sun-4B_Janz*	Singh et al 2013, Agenbag et al 2012, Zwart et al 2010
5A	*IWA2145*	T/C		19.7	0.26	2.1	**4.3**	2	ns	_	*_*	_
5A	*IWA1258*	T/C		235.1	0.29	**3.9**	2	ns	*	MTV14	*QYr*.*cim-5AL_Pastor*, *QYr-5A_Opata_85*	Rosewarne et al 2012, Boukhatem et al 2002
5B	*IWA7815*	A/G	*IWA2335*, *IWA4280*, *IWA6112*	167.8	0.11	**16.7**	**6.4**	3.2	ns	_	*QYr-5B_Oligoculm*, *YrEXP2*	Suenaga et al 2003, McIntosh et al., 2013
6A	*IWA2129*	A/G		212.2	0.05	3.5	**6.9**	ns	***	MTV14, PLM14	*YrLM168*	Rosewarne et al 2012
7B	*IWA312*	A/G	*IWA8300*	76.1	0.08	2.6	**4.8**	**9**	ns	_		
7B	*IWA2770*	A/C		246.5	0.23	**4.2**	2.8	2.1	****	PLM12, MTV12, MTV14, PLM14	*YrZH84*, *YrC591*, *Yr59*,	Li et al 2006, Xu et al 2014, Zhou et al 2014b

^1^SNP index from the wheat 9K iSelect assay.

^2^Underline indicates favorable allele.

^3^Other significant SNPs identified within the confidence interval of the significant QTL.

^4^Based on the consensus map of the wheat 9K iSelect assay by Cavanagh et al. [26].

^5^*P* values-Boldface indicates significance at FDR adjusted probability < 0.1.

MAF—Minor allele frequency.

*, **, *** and **** indicate probability of significance at marker-wise *P* values of < 0.05, 0.01, 0.005 and 0.0001, respectively, in at least two test environments.

PLM12 = Pullman 2012, PLM14 = Pullman 2014, MTV12 = Mount Vernon 2012, MTV14 = Mount Vernon 2014.

### Relationship between number of favorable alleles and response to stripe rust

**Fig 6:**
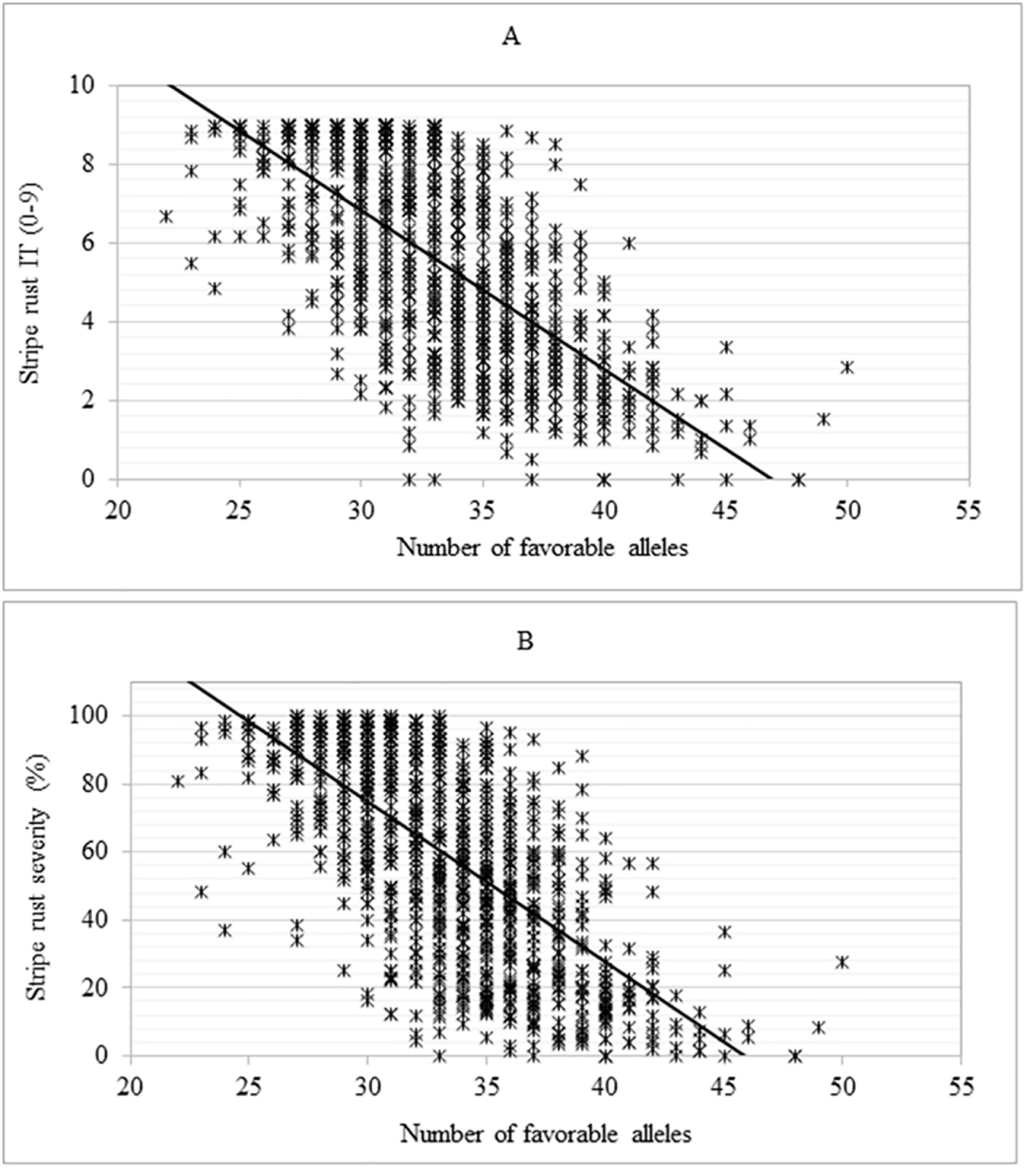
Relationships between the number of favorable alleles of the significant SNPs in each of the accessions and responses to *Puccinia striiformis* f. sp. *tritici*. (A) Infection type (IT), (B) Disease severity (SEV).

The number of favorable alleles of the SNPs representing the 70 genomic regions in each of the 959 accessions varied from 22–50. When accessions were sorted in descending order by the number of favorable alleles, stripe rust SEV and IT in the top 10% accessions (with average number of favorable alleles of 41) were 65.1 and 62.0% lower than the bottom 10% accessions (with average number of favorable alleles of 26). We investigated the relevance of the addition of every favorable allele in an accession in predicting the accessions’ stripe rust response (their additive effect to enhance *Pst* resistance) by correlating BLUP values of IT and SEV on the cumulative number of beneficial alleles of the accessions. There was a highly significant negative correlation (*P* <0.0001, *r* = 0.67) between the number of favorable alleles in individual accessions and the respective stripe rust IT and SEV (**Fig 6**).

### Effect of previously mapped *Yr* genes

Allelic frequencies of markers linked to *Lr67/Yr46*, *Lr34/Yr18*, *Yr17*, *Yr30* and the leaf rust resistance gene *Lr68* are presented in **S2 Fig**. The *Lr34/Yr18*-diagnostic marker was present in 8.5% of the accessions. The resistant allele of this locus reduced IT and SEV by 1.5 (25%) and 18 (32%), respectively. Similarly, association analysis in which the *Lr34* KASP marker data was included detected a strong association of the *Lr34/Yr18* locus with reactions to *Pst* (FDR adjusted *P* value < 0.1) at multiple environments. No significant SNP markers were detected in the 7D region where *Lr34/Yr18* resides. Likewise, none of the SNPs on chromosome 7D were in significant LD with wMAS000003, indicating the lack of significant MTAs on 7D in this study was due to inadequate marker coverage of the chromosome.

The minor allele frequency of the marker *Kasp856* (tightly linked to the *Yr* gene *Lr67/Yr46*) in this germplasm panel was 7.8%. The *Yr46* gene reduced IT and SEV by 2.3 (38%) and 24.6 (44%), and showed strong association with *Pst* resistance (FDR adjusted *P* value < 0.1) in multiple environments. Similar to the report by Maccaferri et al. [17] and Forrest et al. [27], we also observed that *Kasp856* is in LD with *IWA5707* (*r*^*2*^ = 0.40) and other associated SNPs within the confidence interval of the QTL tagged by *IWA5707* (*IWA6277*, *IWA5375* and *IWA5766*) (*r*^*2*^ = 0.31, 0.33 and 0.39, respectively).

*Yr30/Sr2* was present in about 4% of the accessions (**S2 Fig**) that were collected largely from countries from South America. The resistance-associated allele of the marker for *Yr30/Sr2* reduced stripe rust by an average of 12.1%. Since the minor allele frequency of the *Yr30/Sr2* marker was less than 5%, it was excluded from the genome-wide association analyses.

In order to determine whether the slow rusting leaf rust resistance gene, *Lr68*, influences reaction to *Pst*, we performed GWAS analyses and calculated allelic effects associated with the marker closely linked to *Lr68* (cs7BLNLRR). The marker had an effect of reducing stripe rust IT and SEV by 1.5 (25%) and 15 (27%), respectively. Similarly, GWAS showed significant association of the cs7BLNLRR with reactions to *Pst* (marker-wise *P* <0.01) in three of the five environments. Further genetic analysis is required to determine whether the observed allelic effect is conferred by *Lr68* itself or another linked stripe rust gene/QTL.

## Discussion

### Response of spring wheat accessions to stripe rust

The limited availability of well-characterized and effective sources of stripe rust resistance in the elite wheat germplasm pool has constrained the capacity for routine development of varieties with durable resistance. Identifying new sources of resistance and incorporating them into adapted cultivars is therefore a pressing priority to sustainably enhance stripe rust resistance breeding. The present study characterized a large and diverse sample of the NSGC spring wheat core collection for seedling and field-based resistance to the prevailing populations of *Pst* in the Pacific Northwest of the US. These accessions exhibit considerable variation for both seedling and field-based resistance to *Pst*. Among the 464 accessions that showed strong to moderate resistance, with IT ratings <5, 387 accessions were resistant only at post-seedling stage of growth. This may indicate that the resistance in these accessions is likely conferred by APR genes/QTL. Mining of the useful stripe rust resistance genes in such germplasm resources and incorporating them into breeding programs should enhance the durability of released cultivars and mitigate losses due to the disease.

### Population structure of the spring wheat accessions

In the present study, population structure of the 959 accessions from the spring wheat core collection was used as covariate in the GWAS analysis to reduce the likelihood of false positive associations. Structure analysis was also used to describe the effect of geographic origin and improvement status of the accessions in generating patterns of adaptive structure and clustering related to stripe rust resistance. The model based clustering algorithm of structure analysis and the distance-based measure of cryptic relatedness revealed the clustering of the panel into two major subpopulations and discovered a substantial agreement between the patterns of population sub-clustering and information of geographic origin and accessions types of the NSGC spring wheat core collection. The two sub-populations reveal a major division between accessions from Europe, North America and South America (sub-population 1) and accessions from Asia (sub-population 2) and grouping between landrace lines and advanced breeding lines and cultivars. This pattern of population clustering and genetic relatedness among the spring wheat accessions may reflect the impact of unique selection pressures and adaptation of the accessions in each of the diverse environments during the process of domestication and expansion of cultivated *T*. *aestivum* [14,39].

### Marker-trait association

GWAS of stripe rust resistance using the high-density molecular marker information and *Pst* resistance data from multi-environmental field trials and greenhouse experiments provided a basis for comprehensive analysis of the NSGC spring wheat core collection. A total of 70 genomic regions significant at marker-wise *P* <0.01 in at least two of the five environments were identified that were retained in the stepwise regression model. Considering the experiment-wise correction for multiple testing (FDR adjusted *P* <0.1), 11 of the 70 genomic regions were declared significant for field-based resistance to stripe rust. Seven additional regions were significant at FDR *P* <0.1 for seedling resistance. The 11 high-confidence genomic regions explained on average 24% of the total variation in stripe rust IT and SEV; the trait variation explained by the 70 marker-wise significant SNPs was 52%. Hence, although the 70 SNP loci may exhibit some proportion of false positives, the increase in the amount of variation explained by the 70 genomic regions from 24% to 52% suggests the presence of additional true associations. The highly significant negative correlation (*P* <0.0001, *r* = 0.67) between the number of favorable alleles in individual accessions and the respective stripe rust IT and SEV also support the above hypothesis.

### Alignment of the putative QTL to previously mapped *Yr* genes/QTL

The integrated genetic map information constructed by Maccaferri et al. [17] was used to compare resistance loci detected in the current study with previously mapped *Yr* genes/ QTL. Three of the genomic regions that were significant at FDR <0.10, *IWA1755* and *IWA7306* from the field experiments and *IWA2194* from the seedling screening, were mapped far from the previously identified stripe rust resistance genes and QTL. Hence, these three genomic regions most likely tag new stripe rust resistance loci. The genomic regions associated with the remaining 9 putative QTL from the field experiments and six loci from the seedling resistance screening were mapped close to known stripe rust resistance genes and QTL. The relationship of the 15 significant genomic regions with previously mapped *Yr* genes and QTL are discussed below.

#### Chromosome 1B

SNP *IWA1191* was mapped to the proximity of several previously mapped *Yr* genes on the short arm of chromosome 1B. The genetic map position of *IWA1191* (23.7 cM) was at a relative genetic distance of 3.1–6.8, 4.6–6.3, 4.6–14, 6.3–7.7 and 5.9–23.4 cM from *YrAlp*, *Yr15*, *Yr64*, *YrCH52* and *Yr24/Yr26* [40–43]. Evidently, *IWA1191* represents a genomic region associated with a resistance locus that was not effective against any of the three *Pst* races at seedling stage, indicating that the associated locus is likely an APR QTL. However, all of the *Yr* genes previously mapped in this region of 1BS confer major gene or all-stage resistance to stripe rust. Hence, the genomic region tagged by SNP *IWA1191* is likely linked to a different QTL conferring effectiveness to stripe rust at post seedling stage. Another temporarily designated stripe rust APR QTL, *QYr*.*caas-1BL*.*1RS_SHA3/CBRD*, was also mapped close to the region of *IWA1191* (4.6–6.3 cM) [44], which could be related to *IWA1191*.

SNP *IWA5861* was mapped at 70.5 cM proximal to *IWA1191*. Based on its genetic map position, it is unlikely that *IWA5861* is related to *YrAlp*, *Yr15*, *Yr64*, *YrCH52* and *Yr24/Yr26* stripe rust resistance genes. Yet, several other temporarily designated QTL have been identified within the confidence interval of the QTL represented by *IWA5861*. These include *QYr*.*sun-1B_CPI133872*, *QYr*.*sun-1B_Kukri*, *QYr*.*sun-1B_Wollaroi*, *QYr*.*cim-1BL_Francolin*, *QYr*.*tam-1B_Quaiu* and *QYr-1B_Saar* [45–49]. Allelism tests will be required to determine the genetic relationship of the two resistance loci linked to the genomic region represented by *IWA5861* and *IWA1191* with previously mapped QTL. The position of *IWA5861* also overlaps with the race-specific gene *YrExp1* [50]. However, the current races of *Pst* in the Pacific Northwest region are virulent on *YrExp1*. Hence, *IWA5861* is most likely different from *YrExp1*. The position of *IWA5861* is also close to *IWA3017* and *IWA5915*, which showed significant association in studies by Maccaferri et al. [17] and Bulli et al. [22], respectively. In the present wheat population, LD between *IWA5861* and *IWA3017* was determined to be 0.50, indicating that the two SNPs are tagging the same putative stripe rust resistance locus. In the study by Maccaferri et al. [17], *IWA3017* showed only a marker-wise level of association with stripe rust resistance. The strong association of *IWA5861* in the present study (FDR <0.1) confirms that *IWA5861* and *IWA3017* tag a validated QTL. However, LD between *IWA5861* and *IWA5915* is less than 0.002, indicating that the two SNPs are putatively linked to two different stripe rust resistance QTL.

#### Chromosome 2B

*IWA3621* was mapped on chromosome 2B at 114.3 cM, which is in the confidence interval of several previously identified stripe rust QTL. These QTL include *YrKK*, *QYr*.*inra-2B*.*1_Camp Remy*, *QYr*.*ucw-2B_UC1110*, *QYr*.*tam-2BL2_TAM111*, *QYr-2B_Opata 85*, *Qyrlo*.*wpg-2B_Louise* and *QYrid*.*ui-2B*.*2_IDO444* [51–56]. It is therefore likely that *IWA3621* represents a genomic region associated to either one or more of these QTL. On the long arm of 2B, *IWA1040* mapped (210.1 cM) close to the region of *Yr53* (5–11 cM) and *Yr43* (9–18 cM) [57,58]. *Yr53* confers major gene resistance to stripe rust that was derived from durum wheat. *Yr43* is a single dominant gene conferring race-specific all-stage resistance to stripe rust. However, *IWA1040* is likely linked to an APR QTL as it was ineffective at seedling stage and hence may represent a different QTL from the two genes. Another QTL, *QYraq*.*cau-2BL_Aquileja*, was mapped near *IWA1040* by Guo et al. [59], which requires further genetic analysis to determine their relationship.

#### Chromosome 3B

*IWA6843* was mapped at 111.6 cM on chromosome 3B. Although no officially designated *Yr* genes have been reported near this locus, two temporarily designated stripe rust QTL were previously mapped within the confidence interval of the QTL tagged by *IWA6843*. These QTL are *QYr*.*cim-3B_Pastor* [60] and *QYr*.*inra-3Bcentr_Renan* [61]. Further genetic analysis will be required to determine the relationship between *IWA6843* and the two previously mapped QTL.

#### Chromosome 4B

*IWA2194* was significantly associated with resistance against races PSTv-14, PSTv-37 and PSTv-40, as well under field conditions at marker-wise level of probability < 0.01. It was mapped to the proximity of a previously mapped QTL, *QYr*.*ufs-4B_Palmiet* [62]. However, *QYr*.*ufs-4B_Palmiet* is linked to a minor effect APR QTL unlike *IWA2194*, which tags a major effect seedling resistance to *Pst*. Hence, it is likely that *IWA2194* and *QYr*.*ufs-4B_Palmiet* are different.

#### Chromosome 4D

On the short arm of chromosome 4D, we detected a significant association of *IWA5707* and other linked SNPs (*IWA6277*, *IWA5375* and *IWA5766*). Forrest et al. [27] reported an identification of significant association of *IWA5707*, *IWA5375* and *IWA5766* with resistance to stripe rust. Similarly, Maccaferri et al. (2015) also reported a significant association of *IWA5375* and another SNP (*IWA5766*) linked with stripe rust resistance. Based on moderate to strong LD between *IWA5707*, *IWA5766* and the KASP marker *csSNP856*, Forrest et al. [27] determined that these markers are linked to the *Lr67/Yr46* locus. The identification of such previously reported significant marker-trait association further validates our current GWAS analysis.

#### Chromosome 5A

Two SNP loci were significantly associated with seedling resistance to *Pst* races; *IWA2145*, mapped at 19.7 cM, and *IWA1258* mapped at 235.1 cM. *IWA2145* is likely linked to a new *Pst* resistance locus as there are no previously mapped *Yr* genes or QTL close to this locus. *IWA1258* is mapped close (4.3–25.0 cM) to two previously mapped QTL. These are *QYr*.*cim-5AL_Pastor* [60] and *QYr-5A_Opata_85* [55]. Both are minor QTL for APR, unlike *IWA1258*, which likely tags a major effect seedling resistance gene.

#### Chromosome 5B

On 5BL, *IWA7815* and several other associated SNPs represent a locus most strongly associated with seedling resistance to all three *Pst* races. This locus was not effective under field conditions; possibly, due to the presence of other virulent races than the three used in this study. Other QTL including *QYr-5B_Oligoculm* [63] and *YrEXP2* [64] were previously mapped close to the region of *IWA7815*. *QYr-5B_Oligoculm* represents a minor gene-based APR, while *YrExp2* is a seedling resistance gene effective against some *Pst* races, but ineffective to the three races used in our GWAS analysis, indicating that the *IWA7815* tags a locus different from the two previously mapped QTL.

#### Chromosome 6A

On 6AL, we identified SNP *IWA2129* that was significant for seedling resistance to PSTv-14 and PSTv-37, and effective under field conditions at a threshold of marker-wise *P* <0.01 in at least two of the five environments. Its effectiveness both at seedling and adult plant stages may indicate that it is a major seedling resistance gene effective against multiple races of the pathogen. Other *Yr* genes and QTL that have been previously reported close to *IWA2129* include *YrLM168* [65], *QYr-6A_Avocet* [66], *QYr-6A_Saar* [49], *QYr*.*ufs-6A_Kariega* [67] and *QYr*.*cim-6AL_Francolin* [63], which requires further genetic analysis to determine the relationship between the gene tagged by *IWA2129* and previously identified QTL.

#### Chromosome 6D

SNP *IWA167*, mapped on the short arm of 6D at 16.8 cM, is one of the 11 strongly associated genomic regions and has previously been reported by Maccaferri et al. [17]. Detailed information on the *IWA167* locus can be referenced from the previous study [17].

#### Chromosome 7A

Two stripe rust resistance regions were identified on chromosome 7A; *IWA7306* at 6.2 cM, and *IWA1845* at 42.5 cM, Rosewarne et al. [60] and Crossa et al. [68] reported a temporarily designated QTL (*QYr*.*cim-7AS_Avocet* and *QYr*.*cim_7A*.*1_GWAS*, respectively), which were mapped in close proximity to the *IWA7306* locus. Similarly, Zwart et al. [45] and Ren et al. [44] reported two QTL, *QYr*.*sun-7A_CPI133872* and *QYr*.*caas-7A_Jingshuan16*, that were mapped close to *IWA1845*. The identity or similarity of the genomic region tagged by *IWA7306* and *IWA1845* with previously mapped QTL needs to be further investigated. Although *Yr61* [69] and *Yrxy1* [70] have already been mapped on the short arm of chromosome 7A, both *IWA7306* and *IWA1845* were mapped far from *Yr61* and *Yrxy1*, and likely represent different resistance loci.

#### Chromosome 7B

On the long arm of chromosome 7B, SNP *IWA3415* was effective across four field trials and effective at the seedling stage. It was mapped to the vicinity of *Yr59*, *YrC591*, *Yr52*, *Yr67* and *YrZH84* [40,71–75]. *Yr52* and *Yr59* confer high-temperature adult-plant (HTAP) resistance, while *YrC591*, *Yr67* and *YrZH84* confer seedling resistance to stripe rust. *IWA3415* is likely more related to the latter three *Yr* genes due to its effect at a seedling stage. On chromosome 7B *IWA312* (76.1 cM) and *IWA2770* (151.5 cM) were also significant for seedling resistance at experiment-wise threshold of *P* <0.1. *IWA312* could be related to the previously mapped stripe rust *QTL QYr-7B_Oligoculm* [63], but further genetic analysis will be required to determine their relationship. *IWA2770* was also significant under field conditions at marker-wise *P* <0.01 across multiple environments. LD *r*^*2*^ between *IWA3415* and *IWA2770* was 0.03, which indicates that they likely represent different loci. Allelism tests will be required to determine the relationship between the significant SNP loci on 7B and the previously mapped genes on this chromosome.

## Conclusions

The results of the present studies emphasize the prospect of exploiting the high genetic diversity and extensive LD due to historical recombination in wheat germplasm collections to identify genomic regions underpinning resistance to stripe rust. The USDA NSGC spring wheat accessions exhibited a wide range of phenotypic diversity for field-based and seedling resistance to stripe rust. Accessions with a higher percentage of stripe rust resistance-associated alleles are valuable genetic resources that could serve as parental breeding lines to enable more efficient breeding for stripe rust resistance. The molecular markers linked to QTL identified in the current GWAS studies will be of considerable interest for marker-assisted selection in wheat breeding. These genomic regions provide the initial step towards a quantitative, methodical exploitation of untapped genetic diversity in germplasm collection for wheat improvement. Allelism tests will be required to validate these QTL by using bi-parental populations or near-isogenic lines (NILs) and to determine which of the identified QTL represent novel resistance genes and which ones are alleles of previously mapped genes.

## Supporting information

S1 Fig Estimation of the most likely number of clusters in the germplasm panel based on the Bayesian clustering model in structure software. Mean of Estimated Log-likelihood and the magnitude of delta K (*ΔK*) were plotted against K values based on five independent runs and K ranging from 1 to 10.
(PPTX)

S2 Fig Allelic frequency of the markers linked to previously mapped stripe rust resistance genes in the spring wheat association mapping panel. (PPTX)

S1 Table Virulence/avirulence formula of the stripe rust isolates used for seedling resistance screening. (DOCX)

S2 Table Pearson’s correlation coefficients between the five test environments for stripe rust infection types (IT) and severity (SEV). (DOCX)

S3 Table SNP markers tagging QTL for resistance to stripe rust under field condition in a global collection of spring wheat accessions identified based on marker-wise *P* value < 0.01 in at least two environments for both IT and SEV in the GWAS test. (DOCX)

S4 Table Summary of stepwise regression analysis. Parameter estimate, reduction in the residual sum of squares (SS), test statistic and significance level are reported.
(DOCX)
