# Supplementary material for: Loci associated with resistance to stripe rust (Puccinia striiformis f. sp. tritici) in a core collection of spring wheat (Triticum aestivum)
Source: PLoS One. 2017 Jun 7;12(6):e0179087. doi: 10.1371/journal.pone.0179087 (PMC5462451; doi:10.1371/journal.pone.0179087)
Supplement: S1 Fig — Mean of Estimated Log-likelihood and the magnitude of delta K (ΔK) were plotted against K values based on five independent runs and K ranging from 1 to 10. (PPTX) [file pone.0179087.s001.pptx]

## Slide 1
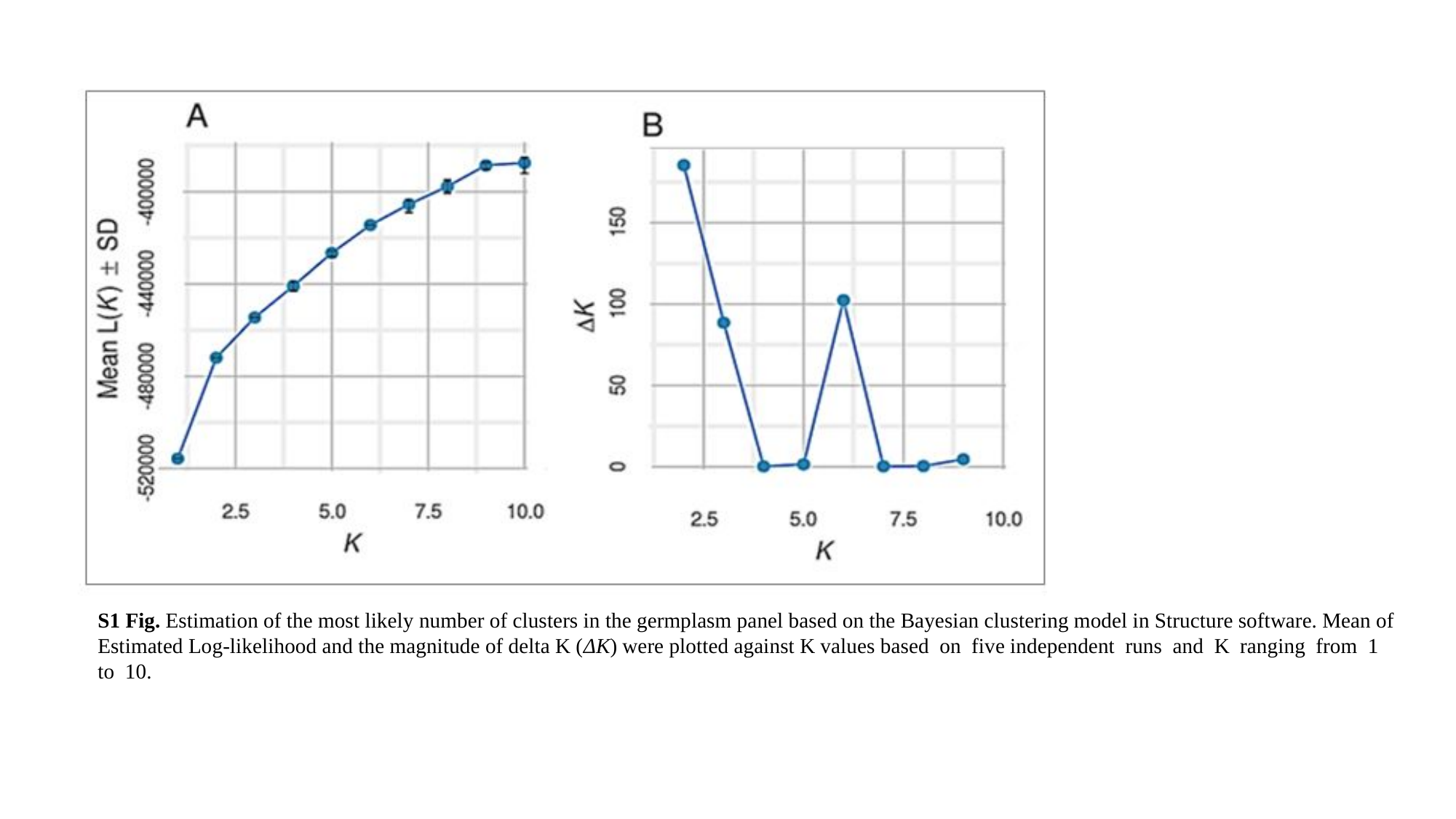

S1 Fig. Estimation of the most likely number of clusters in the germplasm panel based on the Bayesian clustering model in Structure software. Mean of Estimated Log-likelihood and the magnitude of delta K (ΔK) were plotted against K values based on five independent runs and K ranging from 1 to 10.
