# Supplementary material for: Loci associated with resistance to stripe rust (Puccinia striiformis f. sp. tritici) in a core collection of spring wheat (Triticum aestivum)
Source: PLoS One. 2017 Jun 7;12(6):e0179087. doi: 10.1371/journal.pone.0179087 (PMC5462451; doi:10.1371/journal.pone.0179087)
Supplement: S2 Fig — (PPTX) [file pone.0179087.s002.pptx]

## Slide 1
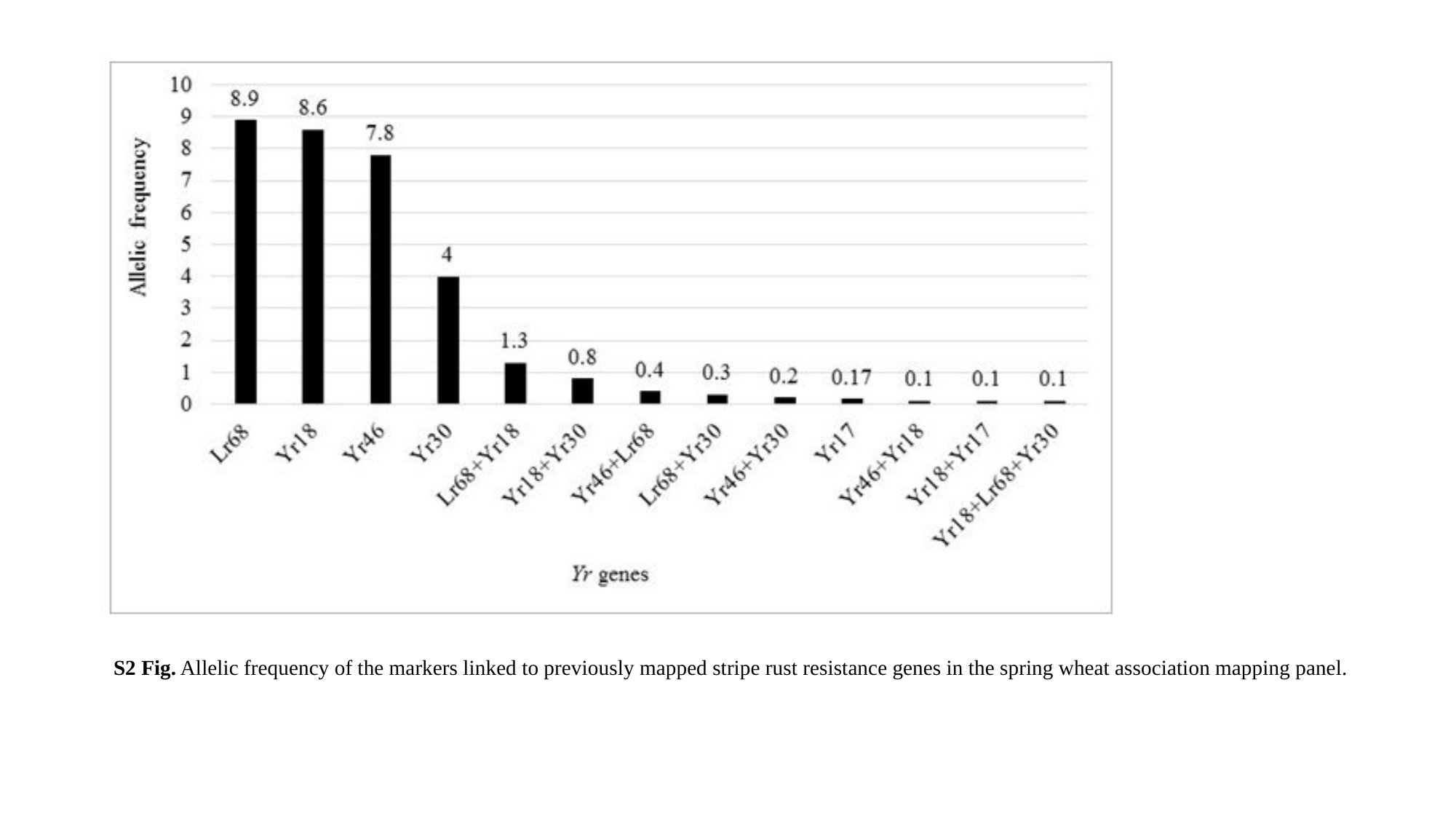

S2 Fig. Allelic frequency of the markers linked to previously mapped stripe rust resistance genes in the spring wheat association mapping panel.
