# Supplementary material for: Loci associated with resistance to stripe rust (Puccinia striiformis f. sp. tritici) in a core collection of spring wheat (Triticum aestivum)
Source: PLoS One. 2017 Jun 7;12(6):e0179087. doi: 10.1371/journal.pone.0179087 (PMC5462451; doi:10.1371/journal.pone.0179087)
Supplement: S1 Table — (DOCX) [file pone.0179087.s003.docx]

**S1 Table.** Virulence/avirulence formula of the stripe rust isolates used for seedling resistance screening.

| **Race** | **Virulence /avirulence formula** |
| --- | --- |
| ***PSTv_14*** | ***Yr1*, *Yr6*, *Yr7*, *Yr8*, *Yr9*, *Yr17*, *Yr27*, *Yr43*, *Yr44*, *YrTr1*, *YrExp2*, *YrTye/*** *Yr5*, *Yr10*, *Yr15*, *Yr24*, *Yr32*, *YrSP* |
| ***PSTv_37*** | ***Yr6*, *Yr7*, *Yr8*, *Yr9*, *Yr17*, *Yr27*, *Yr43*, *Yr44*, *YrTr1*, *YrExp2/*** *Yr1*, *Yr5*, *Yr10*, *Yr15*, *Yr24*, *Yr32*, *YrSP*, *YrTye* |
| ***PSTv_40*** | ***Yr1*, *Yr6*, *Yr7*, *Yr8*, *Yr9*, *Yr10*, *Yr24*, *Yr27*, *Yr43*, *Yr44*, *YrTr1*, *YrExp2****/ Yr5*, *Yr10*, *Yr15*, *Yr24*, *Yr32*, *YrSP* |
