# Supplementary material for: Loci associated with resistance to stripe rust (Puccinia striiformis f. sp. tritici) in a core collection of spring wheat (Triticum aestivum)
Source: PLoS One. 2017 Jun 7;12(6):e0179087. doi: 10.1371/journal.pone.0179087 (PMC5462451; doi:10.1371/journal.pone.0179087)
Supplement: S2 Table — (DOCX) [file pone.0179087.s004.docx]

**S2 Table**. Pearson’s correlation coefficients between the five test environments for stripe rust infection types (IT) and severity (SEV).

| Stripe rust IT and SEV^ǂ^ | PLM_SEV_12 | PLM_SEV_14 | PLM_IT_12 | PLM_IT_14 | MTV_SEV_12 | MTV_SEV_13 | MTV_SEV_14 | MTV_IT_12 | MTV_IT_13 | MTV_IT_14 |
| --- | --- | --- | --- | --- | --- | --- | --- | --- | --- | --- |
| PLM_SEV_12 |  |  |  |  |  |  |  |  |  |  |
| PLM_SEV_14 | 0.70 |  |  |  |  |  |  |  |  |  |
| PLM_IT_12 | 0.86 | 0.71 |  |  |  |  |  |  |  |  |
| PLM_IT_14 | 0.67 | 0.91 | 0.72 |  |  |  |  |  |  |  |
| MTV_SEV_12 | 0.84 | 0.63 | 0.72 | 0.58 |  |  |  |  |  |  |
| MTV_SEV_13 | 0.80 | 0.71 | 0.73 | 0.65 | 0.75 |  |  |  |  |  |
| MTV_SEV_14 | 0.77 | 0.78 | 0.73 | 0.70 | 0.73 | 0.79 |  |  |  |  |
| MTV_IT_12 | 0.78 | 0.68 | 0.85 | 0.67 | 0.88 | 0.71 | 0.71 |  |  |  |
| MTV_IT_13 | 0.77 | 0.70 | 0.76 | 0.68 | 0.74 | 0.92 | 0.76 | 0.74 |  |  |
| MTV_IT_14 | 0.73 | 0.77 | 0.75 | 0.75 | 0.66 | 0.72 | 0.89 | 0.71 | 0.72 |  |

^ǂ^All correlation coefficients are highly significant (*P<* 0.0001).

PLM_SEV_12 = Pullman, Severity, 2012

PLM_SEV_14 = Pullman, Severity, 2014

PLM_IT_12 = Pullman, Infection Type, 2012

PLM_IT_14 = Pullman, Severity, 2014

MTV_SEV_12 = Mount Vernon, Severity, 2012

MTV_SEV_13 = Mount Vernon, Severity, 2013

MTV_SEV_14 = Mount Vernon, Severity, 2014

MTV_IT_12 = Mount Vernon, Infection Type, 2012

MTV_IT_13 = Mount Vernon, Infection Type, 2013

MTV_IT_14 = Mount Vernon, Infection Type, 2014
