# Supplementary material for: Loci associated with resistance to stripe rust (Puccinia striiformis f. sp. tritici) in a core collection of spring wheat (Triticum aestivum)
Source: PLoS One. 2017 Jun 7;12(6):e0179087. doi: 10.1371/journal.pone.0179087 (PMC5462451; doi:10.1371/journal.pone.0179087)
Supplement: S3 Table — (DOCX) [file pone.0179087.s005.docx]

**S3 Table.** SNP markers tagging QTL for resistance to stripe rust under field condition in a global collection of spring wheat accessions identified based on marker-wise *P* value < 0.01 in at least two environments for both IT and SEV in the GWAS test.

| QTL-tagging SNP^1^ | Alleles^2^ | | Chr. | Pos^3^ (cM) | MAF^4^ | Associated SNP^5^ | P values (-log)^6^ | |
| --- | --- | --- | --- | --- | --- | --- | --- | --- |
|  |  |  |  |  |  |  | IT | SEV |
| IWA414 | T | G | 1A | 28.21 | 0.38 |  | 2.31 | 2.57 |
| IWA217 | A | C | 1A | 45.87 | 0.43 |  | 2.09 | 2.59 |
| IWA4327 | A | G | 1A | 75.77 | 0.31 | IWA3857 IWA7862 | 2.38 | 2.26 |
| IWA1593 | A | G | 1A | 83.02 | 0.06 |  | 1.95 | 2.15 |
| IWA8214 | T | G | 1A | 173.72 | 0.23 | IWA8213, IWA7034 | 2.17 | 2.36 |
| IWA4349 | T | C | 1B | 13.19 | 0.30 |  | 1.49 | 2.01 |
| IWA1191 | T | C | 1B | 23.65 | 0.18 |  | 2.32 | 2.63 |
| IWA7214 | T | G | 1B | 66.51 | 0.08 |  | 2.38 | 2.44 |
| IWA5861 | T | C | 1B | 94.18 | 0.23 | IWA3017 | 2.89 | 2.80 |
| IWA4645 | T | C | 1D | 26.29 | 0.49 |  | 2.26 | 2.41 |
| IWA642 | A | G | 1D | 50.75 | 0.37 |  | 1.69 | 1.82 |
| IWA3294 | T | C | 2A | 95.94 | 0.21 | IWA5306, IWA7429, IWA2195 | 2.56 | 2.71 |
| IWA5130 | A | G | 2A | 152.7 | 0.11 |  | 2.16 | 2.07 |
| IWA2092 | T | C | 2A | 160.72 | 0.30 |  | 1.93 | 1.88 |
| IWA2778 | T | G | 2A | 218.99 | 0.27 | IWA5879 | 2.31 | 2.14 |
| IWA652 | T | C | 2B | 110.85 | 0.14 | IWA3277 | 2.19 | 2.40 |
| IWA3621 | A | G | 2B | 114.31 | 0.31 | IWA4984, IWA6875, IWA6818 | 2.64 | 2.47 |
| IWA7253 | A | G | 2B | 149.36 | 0.10 | IWA587 | 1.98 | 2.52 |
| IWA2874 | T | G | 2B | 208.69 | 0.40 |  | 2.54 | 2.17 |
| IWA1040 | A | G | 2B | 210.15 | 0.11 | IWA8266 | 2.92 | 2.67 |
| IWA5081 | A | G | 2B | 230.09 | 0.17 |  | 1.99 | 2.97 |
| IWA3474 | T | G | 2B | 254.66 | 0.45 |  | 2.19 | 2.19 |
| IWA1601 | A | C | 2D | 10.71 | 0.29 | IWA4354, IWA4711 | 2.49 | 2.23 |
| IWA96 | T | C | 2D | 175.64 | 0.24 |  | 2.04 | 2.27 |
| IWA5429 | A | G | 3A | 3.93 | 0.36 |  | 2.49 | 1.84 |
| IWA7696 | A | C | 3A | 123.35 | 0.3 |  | 2.17 | 1.78 |
| IWA4412 | A | C | 3B | 66.39 | 0.3 |  | 1.69 | 2.33 |
| IWA6482 | A | G | 3B | 88.25 | 0.47 |  | 2.38 | 3.25 |
| IWA7512 | A | G | 3B | 91.11 | 0.45 |  | 1.65 | 1.89 |
| IWA6843 | A | G | 3B | 111.60 | 0.05 |  | 2.78 | 3.05 |
| IWA3592 | T | C | 3B | 123.29 | 0.42 |  | 2.58 | 2.43 |
| IWA6002 | A | C | 3B | 129.50 | 0.43 |  | 2.17 | 2.28 |
| IWA8043 | A | G | 3B | 141.57 | 0.27 |  | 2.78 | 2.33 |
| IWA2148 | A | G | 3B | 180.49 | 0.22 |  | 1.81 | 2.36 |
| IWA5559 | A | C | 4A | 2.42 | 0.17 |  | 2.00 | 2.12 |
| IWA2194 | A | C | 4B | 39.19 | 0.36 |  | 2.21 | 2.11 |
| IWA907 | A | C | 4B | 80.00 | 0.05 |  | 2.62 | 2.51 |
| IWA5707 | T | C | 4D | 20.64 | 0.13 | *IWA6277, IWA5375* *IWA5766* | 2.83 | 2.84 |
| IWA6412 | A | C | 5A | 42.82 | 0.46 |  | 2.17 | 2.30 |
| IWA3647 | A | C | 5A | 80.97 | 0.05 | IWA3413 | 2.77 | 2.42 |
| IWA2363 | T | C | 5A | 101.10 | 0.06 |  | 2.61 | 1.54 |
| IWA4648 | A | G | 5A | 119.31 | 0.24 |  | 2.91 | 2.22 |
| IWA2003 | A | G | 5B | 68.27 | 0.42 |  | 1.89 | 2.33 |
| IWA1755 | A | G | 5B | 130.39 | 0.18 | IWA6627 | 2.58 | 2.76 |
| IWA7910 | A | G | 5B | 156.74 | 0.42 |  | 2.26 | 2.88 |
| IWA7493 | A | G | 5B | 172.48 | 0.09 |  | 1.97 | 1.78 |
| IWA1390 | T | C | 5B | 216.61 | 0.10 |  | 2.45 | 2.71 |
| IWA7177 | T | C | 5D | 17.99 | 0.22 |  | 1.55 | 2.20 |
| IWA5466 | T | C | 6A | 78.23 | 0.27 |  | 2.23 | 2.41 |
| IWA8595 | T | C | 6A | 204.49 | 0.34 |  | 2.36 | 2.23 |
| IWA7897 | A | G | 6B | 40.68 | 0.08 |  | 2.47 | 2.34 |
| IWA6467 | T | C | 6B | 48.80 | 0.20 | IWA6466 | 2.08 | 2.92 |
| IWA7574 | T | C | 6B | 75.80 | 0.45 | IWA3917 | 2.06 | 1.54 |
| IWA3796 | T | C | 6B | 81.26 | 0.44 |  | 2.51 | 1.75 |
| IWA7257 | T | G | 6B | 112.30 | 0.19 |  | 2.13 | 2.55 |
| IWA404 | T | C | 6B | 118.54 | 0.37 |  | 2.94 | 2.29 |
| IWA2808 | A | G | 6D | 58.06 | 0.21 | IWA3624 | 2.82 | 2.36 |
| IWA7816 | A | C | 6D | 68.60 | 0.28 |  | 2.57 | 2.78 |
| IWA167 | A | G | 6D | 73.19 | 0.11 |  | 2.65 | 2.89 |
| IWA7306 | A | G | 7A | 6.21 | 0.48 |  | 2.84 | 2.49 |
| IWA1845 | T | C | 7A | 42.48 | 0.34 | IWA2513 | 2.91 | 2.54 |
| IWA2042 | A | G | 7A | 63.61 | 0.11 |  | 2.46 | 2.53 |
| IWA5527 | T | C | 7A | 102.48 | 0.06 |  | 2.56 | 2.97 |
| IWA593 | A | G | 7A | 107.39 | 0.10 |  | 2.60 | 2.35 |
| IWA6735 | A | G | 7A | 193.82 | 0.34 |  | 2.35 | 2.69 |
| IWA5129 | A | G | 7B | 73.33 | 0.38 | IWA5110, IWA130 | 2.70 | 3.21 |
| IWA5597 | T | C | 7B | 149.45 | 0.11 | IWA2770 | 2.67 | 3.37 |
| IWA2770 | A | C | 7B | 151.55 | 0.21 |  | 2.24 | 2.38 |
| IWA3675 | A | G | 7B | 156.37 | 0.33 |  | 2.47 | 2.61 |
| IWA3415 | A | G | 7B | 164.88 | 0.27 | IWA3416 | 3.13 | 3.85 |

^1^SNP indexes from Illumina iSelect 9K wheat assay (Cavanagh et al. 2013) that represented the significant genomic regions.

^2^Allele associated with the resistance response is underlined.

^3^Genetic map position of the significant SNPs according to Cavanagh et al., 2013.

^4^Minor allele frequency of the SNPs.

^5^SNP loci in LD with the representative SNP and significantly associated to the *Pst* response. ^6^Probabilities are reported as -log (*P* value), **IT** and **SEV** –best linear unbiased estimates (BLUPs) of stipe rust infection types and severity across all environments,.

**Bold** and grey highlighted SNPs are those that were significant at FDR adjusted *P* < 0.1.
