# Supplementary material for: Loci associated with resistance to stripe rust (Puccinia striiformis f. sp. tritici) in a core collection of spring wheat (Triticum aestivum)
Source: PLoS One. 2017 Jun 7;12(6):e0179087. doi: 10.1371/journal.pone.0179087 (PMC5462451; doi:10.1371/journal.pone.0179087)
Supplement: S4 Table — Parameter estimate, reduction in the residual sum of squares (SS), test statistic and significance level are reported. (DOCX) [file pone.0179087.s006.docx]

**S4 Table.** Summary of stepwise regression analysis. Parameter estimate, reduction in the residual sum of squares (SS), test statistic and significance level are reported.

| Parameter | Chr. | Stripe rust IT | | | | Stipe rust SEV | | | |
| --- | --- | --- | --- | --- | --- | --- | --- | --- | --- |
|  |  | Estimate | SS | F Ratio | Prob>F | Estimate | SS | F Ratio | Prob>F |
| IWA1593 | 1A | -0.65 | 15.2 | 3.9 | 4.3E-02 | -8.70 | 2878.2 | 5.5 | 1.9E-02 |
| IWA217 | 1A | -0.82 | 23.2 | 7.2 | 2.5E-06 | -11.61 | 2443.1 | 5.8 | 6.0E-10 |
| IWA414 | 1A | -0.86 | 23.8 | 6.3 | 1.9E-07 | -8.75 | 1853.6 | 4.4 | 1.3E-06 |
| IWA4327 | 1A | – | – | – | – | -3.55 | 1781.2 | 3.7 | 3.7E-02 |
| IWA8214 | 1A | -0.68 | 22.1 | 5.7 | 2.6E-03 | -9.32 | 4678.1 | 8.9 | 1.6E-04 |
| IWA1191 | 1B | -0.91 | 18.8 | 4.8 | 1.2E-05 | -9.90 | 1561.2 | 3.2 | 1.2E-05 |
| IWA4349 | 1B | -0.53 | 13.4 | 4.4 | 2.6E-03 | -5.41 | 1574.1 | 3.5 | 5.0E-03 |
| IWA5861 | 1B | -0.43 | 18.7 | 4.8 | 2.9E-02 | -3.86 | 1538.7 | 3.6 | 5.7E-02 |
| IWA7214 | 1B | -0.83 | 13.6 | 4.3 | 5.2E-03 | -10.55 | 2126.7 | 5.0 | 3.8E-03 |
| IWA4645 | 1D | -0.37 | 17.2 | 4.4 | 3.6E-02 | -3.13 | 1188.1 | 2.8 | 9.4E-02 |
| IWA642 | 1D | -1.02 | 97.7 | 31.0 | 6.1E-10 | -12.48 | 10997.7 | 26.3 | 1.1E-10 |
| IWA2092 | 2A | 0.28 | 9.9 | 3.8 | 4.1E-03 | 4.15 | 2020.6 | 4.2 | 3.1E-04 |
| IWA2778 | 2A | -0.87 | 63.8 | 16.3 | 9.8E-07 | -7.88 | 4441.3 | 8.8 | 4.0E-04 |
| IWA3294 | 2A | -0.59 | 11.6 | 4.4 | 3.6E-03 | -7.73 | 1025.7 | 2.2 | 3.2E-03 |
| IWA5130 | 2A | -0.44 | 11.4 | 3.8 | 5.3E-02 | -5.74 | 1485.2 | 3.6 | 4.6E-02 |
| IWA1040 | 2B | -0.68 | 8.8 | 2.7 | 8.9E-03 | -10.42 | 1634.4 | 3.2 | 1.1E-03 |
| IWA2874 | 2B | -0.68 | 19.2 | 7.3 | 5.9E-05 | -5.06 | 2528.0 | 5.0 | 2.7E-03 |
| IWA3474 | 2B | -0.48 | 9.5 | 2.9 | 5.4E-03 | – | – | – | – |
| IWA3621 | 2B | -1.75 | 16.1 | 5.0 | 4.0E-03 | -6.60 | 4677.3 | 9.7 | 1.9E-03 |
| IWA5081 | 2B | -0.93 | 16.4 | 6.2 | 8.3E-07 | -9.09 | 1225.3 | 2.9 | 2.3E-05 |
| IWA652 | 2B | -1.26 | 10.9 | 2.6 | 2.4E-08 | -16.02 | 1787.1 | 3.7 | 9.0E-09 |
| IWA7253 | 2B | -0.48 | 7.9 | 3.0 | 8.4E-02 | -9.16 | 2727.9 | 5.2 | 2.3E-02 |
| IWA1601 | 2D | -0.62 | 12.0 | 4.5 | 5.6E-05 | -11.00 | 2850.6 | 6.8 | 1.8E-03 |
| IWA96 | 2D | -0.56 | 15.6 | 4.0 | 3.6E-03 | -8.64 | 4062.7 | 8.0 | 3.4E-05 |
| IWA5429 | 3A | -0.30 | 13.7 | 3.5 | 6.1E-02 | -4.89 | 1781.3 | 3.8 | 6.1E-03 |
| IWA7696 | 3A | -0.61 | 20.0 | 6.8 | 4.9E-04 | -5.65 | 1865.6 | 4.0 | 4.7E-03 |
| IWA2148 | 3B | -0.48 | 19.9 | 4.8 | 2.9E-02 | -6.76 | 3908.4 | 7.7 | 5.6E-03 |
| IWA3592 | 3B | -0.61 | 28.4 | 6.8 | 8.8E-05 | -6.01 | 2204.1 | 4.4 | 1.9E-03 |
| IWA4412 | 3B | – | – | – | – | -5.23 | 1897.7 | 3.9 | 9.9E-03 |
| IWA6002 | 3B | -0.74 | 11.6 | 3.8 | 6.6E-07 | -6.15 | 1924.4 | 3.9 | 7.8E-04 |
| IWA6482 | 3B | -0.44 | 13.9 | 4.4 | 1.7E-02 | -3.74 | 781.4 | 1.5 | 3.6E-02 |
| IWA6843 | 3B | -1.48 | 13.4 | 3.4 | 7.1E-06 | -15.34 | 1917.3 | 4.1 | 2.3E-04 |
| IWA7512 | 3B | -0.71 | 17.6 | 4.5 | 6.7E-05 | -7.81 | 4455.3 | 8.5 | 4.8E-05 |
| IWA8043 | 3B | -0.40 | 13.8 | 4.3 | 3.4E-02 | -5.70 | 3503.1 | 7.3 | 7.2E-03 |
| IWA5559 | 4A | -0.68 | 11.4 | 2.7 | 8.3E-04 | -8.44 | 1876.4 | 3.7 | 4.9E-04 |
| IWA2194 | 4B | -0.45 | 26.9 | 6.9 | 8.8E-03 | -4.50 | 2738.0 | 5.2 | 2.2E-02 |
| IWA907 | 4B | -1.02 | 19.4 | 5.0 | 2.4E-03 | -12.52 | 3367.0 | 7.2 | 8.7E-04 |
| IWA5707 | 4D | -1.87 | 6.3 | 2.4 | 7.9E-08 | -15.74 | 1494.3 | 3.5 | 4.4E-05 |
| IWA2363 | 5A | -0.75 | 15.0 | 4.6 | 1.4E-02 | -9.14 | 3154.3 | 7.5 | 6.2E-03 |
| IWA3647 | 5A | -0.89 | 19.9 | 7.5 | 6.3E-03 | -14.80 | 830.7 | 1.7 | 2.6E-04 |
| IWA4648 | 5A | -0.43 | 20.6 | 5.3 | 2.2E-02 | -4.32 | 2134.1 | 4.1 | 4.4E-02 |
| IWA6412 | 5A | 0.30 | 9.3 | 2.4 | 2.0E-05 | 5.68 | 3607.7 | 7.8 | 1.8E-05 |
| IWA1390 | 5B | -0.57 | 19.7 | 5.0 | 2.5E-02 | -8.27 | 4104.7 | 7.8 | 5.2E-03 |
| IWA1755 | 5B | -0.66 | 7.9 | 2.9 | 2.1E-03 | -8.74 | 2946.8 | 6.1 | 2.8E-04 |
| IWA2003 | 5B | -0.49 | 16.3 | 6.2 | 6.9E-03 | -6.16 | 2313.8 | 5.0 | 1.0E-03 |
| IWA7493 | 5B | -1.01 | 11.0 | 3.4 | 4.0E-04 | -13.87 | 2008.1 | 4.0 | 2.7E-05 |
| IWA7910 | 5B | -0.65 | 11.5 | 4.3 | 8.2E-06 | -6.56 | 2120.7 | 4.6 | 9.0E-05 |
| IWA7177 | 5D | -0.73 | 14.0 | 4.3 | 2.7E-04 | -10.12 | 1482.0 | 3.2 | 8.6E-06 |
| IWA5466 | 6A | -0.59 | 20.8 | 5.4 | 4.8E-04 | -7.75 | 4079.7 | 9.8 | 2.6E-04 |
| IWA8595 | 6A | -0.40 | 22.0 | 5.6 | 1.8E-02 | -6.52 | 3218.4 | 7.7 | 4.9E-04 |
| IWA3796 | 6B | -0.46 | 12.1 | 3.1 | 2.3E-03 | -3.88 | 2083.7 | 4.3 | 2.6E-02 |
| IWA404 | 6B | -0.80 | 15.1 | 5.7 | 8.1E-08 | -11.14 | 2003.3 | 3.8 | 7.2E-09 |
| IWA6467 | 6B | -0.69 | 16.2 | 5.3 | 3.1E-04 | -6.96 | 1389.9 | 2.9 | 2.4E-03 |
| IWA7257 | 6B | -0.75 | 14.8 | 5.6 | 5.3E-04 | -6.90 | 3453.0 | 7.7 | 4.4E-03 |
| IWA7574 | 6B | – | – | – | – | 3.68 | 1950.8 | 4.0 | 4.5E-02 |
| IWA7897 | 6B | -1.12 | 18.8 | 5.7 | 5.2E-05 | -13.27 | 4154.7 | 8.6 | 3.5E-05 |
| IWA167 | 6D | -1.70 | 42.1 | 10.2 | 4.9E-11 | -20.20 | 8421.8 | 16.1 | 3.2E-11 |
| IWA2808 | 6D | -0.55 | 24.9 | 6.4 | 1.2E-02 | -5.69 | 1261.6 | 3.0 | 2.6E-02 |
| IWA7816 | 6D | -0.60 | 21.4 | 6.6 | 3.8E-04 | -9.85 | 6791.4 | 14.6 | 2.0E-07 |
| IWA1845 | 7A | -0.78 | 20.8 | 7.9 | 1.6E-05 | -8.30 | 1944.3 | 4.2 | 1.8E-05 |
| IWA2042 | 7A | -0.95 | 21.4 | 5.5 | 4.2E-05 | -11.24 | 2186.1 | 4.3 | 3.1E-05 |
| IWA5527 | 7A | -1.21 | 9.9 | 3.3 | 5.7E-05 | -13.60 | 1038.1 | 2.2 | 3.9E-04 |
| IWA593 | 7A | -0.61 | 18.9 | 6.2 | 1.3E-02 | -9.11 | 4338.4 | 9.3 | 2.3E-03 |
| IWA6735 | 7A | -0.65 | 13.8 | 4.5 | 4.9E-06 | -6.85 | 2133.8 | 5.1 | 1.0E-04 |
| IWA7306 | 7A | -0.68 | 9.4 | 2.3 | 1.3E-07 | -8.48 | 2035.1 | 4.9 | 1.6E-07 |
| IWA2770 | 7B | -0.33 | 9.8 | 2.5 | 1.0E-01 | – | – | – | – |
| IWA3415 | 7B | -1.31 | 53.9 | 13.8 | 5.2E-10 | -16.70 | 8180.4 | 19.4 | 3.1E-13 |
| IWA3675 | 7B | -0.67 | 25.4 | 6.5 | 1.8E-05 | -11.09 | 2245.4 | 4.8 | 1.2E-07 |
| IWA5129 | 7B | -5.96 | 25.4 | 6.5 | 5.1E-05 | -68.67 | 2761.7 | 6.5 | 2.2E-05 |
| IWA5597 | 7B | -1.07 | 15.1 | 4.7 | 3.0E-05 | -11.73 | 1722.5 | 4.1 | 1.7E-04 |
